# Does intracytoplasmic sperm injection outperform conventional *in vitro* fertilization in couples without severe male factor infertility? A systematic review and meta-analysis of randomized controlled trials

**DOI:** 10.1093/humrep/deag066

**Published:** 2026-05-22

**Authors:** Kailibinuer Kayimu, Sine Berntsen, Yu Fu, Yi Yuan, Kilian Vomstein, Tian Tian, Fang Liu, Jiayi Gao, Lan N Vuong, Tuong M Ho, Bao G Huynh, Toan D Pham, Vinh Q Dang, Dea Frøding Skipper, Anne Zedeler, Anja Pinborg, David Westergaard, Ben W Mol, Henriette Svarre Nielsen, Nina la Cour Freiesleben, Jie Qiao, Yuanyuan Wang

**Affiliations:** State Key Laboratory of Female Fertility Promotion, Department of Obstetrics and Gynecology, Peking University Third Hospital, Beijing, China; National Clinical Research Center for Obstetrics and Gynecology (Peking University Third Hospital), Beijing, China; Key Laboratory of Assisted Reproduction (Peking University), Ministry of Education, Beijing, China; Beijing Key Laboratory of Collaborative Innovation in Frontier Technologies for Population, Beijing, China; Department of Obstetrics and Gynaecology, The Fertility Clinic, Copenhagen University Hospital Hvidovre, Hvidovre, Denmark; State Key Laboratory of Female Fertility Promotion, Department of Obstetrics and Gynecology, Peking University Third Hospital, Beijing, China; National Clinical Research Center for Obstetrics and Gynecology (Peking University Third Hospital), Beijing, China; Key Laboratory of Assisted Reproduction (Peking University), Ministry of Education, Beijing, China; Beijing Key Laboratory of Collaborative Innovation in Frontier Technologies for Population, Beijing, China; State Key Laboratory of Female Fertility Promotion, Department of Obstetrics and Gynecology, Peking University Third Hospital, Beijing, China; National Clinical Research Center for Obstetrics and Gynecology (Peking University Third Hospital), Beijing, China; Key Laboratory of Assisted Reproduction (Peking University), Ministry of Education, Beijing, China; Beijing Key Laboratory of Collaborative Innovation in Frontier Technologies for Population, Beijing, China; Department of Obstetrics and Gynaecology, The Fertility Clinic, Copenhagen University Hospital Hvidovre, Hvidovre, Denmark; Institute of Clinical Medicine, University of Copenhagen, Faculty of Health and Medical Sciences, Copenhagen, Denmark; State Key Laboratory of Female Fertility Promotion, Department of Obstetrics and Gynecology, Peking University Third Hospital, Beijing, China; National Clinical Research Center for Obstetrics and Gynecology (Peking University Third Hospital), Beijing, China; Key Laboratory of Assisted Reproduction (Peking University), Ministry of Education, Beijing, China; Beijing Key Laboratory of Collaborative Innovation in Frontier Technologies for Population, Beijing, China; State Key Laboratory of Female Fertility Promotion, Department of Obstetrics and Gynecology, Peking University Third Hospital, Beijing, China; National Clinical Research Center for Obstetrics and Gynecology (Peking University Third Hospital), Beijing, China; Key Laboratory of Assisted Reproduction (Peking University), Ministry of Education, Beijing, China; Beijing Key Laboratory of Collaborative Innovation in Frontier Technologies for Population, Beijing, China; State Key Laboratory of Female Fertility Promotion, Department of Obstetrics and Gynecology, Peking University Third Hospital, Beijing, China; National Clinical Research Center for Obstetrics and Gynecology (Peking University Third Hospital), Beijing, China; Key Laboratory of Assisted Reproduction (Peking University), Ministry of Education, Beijing, China; Beijing Key Laboratory of Collaborative Innovation in Frontier Technologies for Population, Beijing, China; Department of Obstetrics and Gynaecology, University of Medicine and Pharmacy at Ho Chi Minh City, Ho Chi Minh City, Vietnam; IVFMD, My Duc Hospital, Ho Chi Minh City, Vietnam; HOPE Research Centre, My Duc Hospital, Ho Chi Minh City, Vietnam; IVFMD, My Duc Hospital, Ho Chi Minh City, Vietnam; HOPE Research Centre, My Duc Hospital, Ho Chi Minh City, Vietnam; HOPE Research Centre, My Duc Hospital, Ho Chi Minh City, Vietnam; HOPE Research Centre, My Duc Hospital, Ho Chi Minh City, Vietnam; School of Preventative Medicine and Public Health, Monash University, Melbourne, VIC, Australia; Department of Obstetrics and Gynaecology, Copenhagen University Hospital Hvidovre, Hvidovre, Denmark; Department of Obstetrics and Gynaecology, The Fertility Clinic, Copenhagen University Hospital Hvidovre, Hvidovre, Denmark; Institute of Clinical Medicine, University of Copenhagen, Faculty of Health and Medical Sciences, Copenhagen, Denmark; Fertility Clinic, Department of Gynecology, Fertility and Obstetrics, Copenhagen University Hospital Rigshospitalet, Copenhagen, Denmark; Department of Obstetrics and Gynaecology, Copenhagen University Hospital Hvidovre, Hvidovre, Denmark; Department of Health Technology, Technical University of Denmark, Lyngby, Denmark; School of Preventative Medicine and Public Health, Monash University, Melbourne, VIC, Australia; Department of Obstetrics and Gynecology, Monash Health, Melbourne, VIC, Australia; Department of Obstetrics and Gynaecology, The Fertility Clinic, Copenhagen University Hospital Hvidovre, Hvidovre, Denmark; Institute of Clinical Medicine, University of Copenhagen, Faculty of Health and Medical Sciences, Copenhagen, Denmark; Department of Obstetrics and Gynaecology, The Fertility Clinic, Copenhagen University Hospital Hvidovre, Hvidovre, Denmark; Institute of Clinical Medicine, University of Copenhagen, Faculty of Health and Medical Sciences, Copenhagen, Denmark; State Key Laboratory of Female Fertility Promotion, Department of Obstetrics and Gynecology, Peking University Third Hospital, Beijing, China; National Clinical Research Center for Obstetrics and Gynecology (Peking University Third Hospital), Beijing, China; Key Laboratory of Assisted Reproduction (Peking University), Ministry of Education, Beijing, China; Beijing Key Laboratory of Collaborative Innovation in Frontier Technologies for Population, Beijing, China; State Key Laboratory of Female Fertility Promotion, Department of Obstetrics and Gynecology, Peking University Third Hospital, Beijing, China; National Clinical Research Center for Obstetrics and Gynecology (Peking University Third Hospital), Beijing, China; Key Laboratory of Assisted Reproduction (Peking University), Ministry of Education, Beijing, China; Beijing Key Laboratory of Collaborative Innovation in Frontier Technologies for Population, Beijing, China

**Keywords:** live birth, conventional IVF, ICSI, systematic review, meta-analysis

## Abstract

**STUDY QUESTION:**

Does ICSI improve the live birth rate in couples without severe male factor infertility compared to conventional IVF (cIVF)?

**SUMMARY ANSWER:**

High-quality evidence showed no benefit of ICSI over cIVF in improving live birth or cumulative live birth rates among couples without severe male factor infertility.

**WHAT IS KNOWN ALREADY:**

Although ICSI is an effective method within ART for severe male factor infertility, it is frequently used for other infertility etiologies despite insufficient evidence. The effectiveness of ICSI compared with cIVF in couples with mild male or without severe male factor infertility remains uncertain.

**STUDY DESIGN, SIZE, DURATION:**

Systematic review and meta-analysis. PubMed, EMBASE, MEDLINE, Web of Science, Cochrane Library, ProQuest Dissertations & Theses Global, Scopus, CINAHL Plus, Chinese Wan Fang, and CNKI databases were searched from inception to 31 May 2025 without language restrictions. The search strategy encompassed three key domains: ICSI, cIVF, and ART treatment outcomes.

**PARTICIPANTS/MATERIALS, SETTING, METHODS:**

We included randomized controlled trials (RCTs) comparing outcomes of ICSI versus cIVF per couple. Exclusion criteria were duplicate studies, conference abstracts or proceedings, trial registry records, editorials, letters, non-randomized designs, RCTs that did not randomize participants to ICSI or cIVF, studies comparing effects per oocyte rather than per couple, studies lacking complete outcome data, and those not meeting predefined criteria for trustworthiness. Study characteristics and ART outcomes were extracted. The risk of bias and study trustworthiness were independently evaluated by two investigators using the Cochrane Collaboration’s Risk of Bias 2 Tool and TRACT checklist, respectively. GRADE decision-making was used to evaluate the quality of evidence.

**MAIN RESULTS AND THE ROLE OF CHANCE:**

Six RCTs reporting on couples without severe male factor infertility were included. The meta-analysis showed no benefit from ICSI over cIVF in live birth rate (four studies, N = 1438, 32.8% vs 34.5%, pooled risk ratio (RR) = 0.96, 95% CI: 0.85–1.09, *I*^2^ = 37%, high-quality evidence) or cumulative live birth rate (three studies, N = 1911, 43.2% vs 47.4%, pooled RR = 0.92, 95% CI: 0.84–1.01, *I*^2^ = 41%, high-quality evidence). ICSI was associated with a lower preterm birth rate (three studies, N = 222, 4.6% vs 6.0%, pooled RR = 0.77, 95% CI: 0.59–1.00, *P* = 0.0447, *I*^2^ = 0, high-quality evidence). No significant differences were observed for other fertility or pregnancy outcomes.

**LIMITATIONS, REASONS FOR CAUTION:**

The findings should be interpreted with caution due to the limited number of high-quality studies reporting live birth data, limited subgroup-specific evidence, and some heterogeneity in outcome measures.

**WIDER IMPLICATIONS OF THE FINDINGS:**

Evidence from this meta-analysis shows no advantage of ICSI over cIVF in improving live birth or cumulative live birth rates among couples without severe male factor infertility. Based on current evidence, ICSI should not be routinely recommended for indications other than severe male infertility.

**STUDY FUNDING/COMPETING INTEREST(S):**

The study was funded by the National Natural Science Foundation of China (No. 82204052), the National Key Research and Development Program of China (No. 2022YFC2703102), and Peking University Third Hospital (No. BYSYDL2022001, BYSYDL2024003) with salaries for J.Q., Y.W., K.K., Y.F., Y.Y., T.T., F.L., and J.G. The funders of the study played no role in study design, data collection, data analysis, data interpretation, or writing of the report. S.B. has received scientific grants from Gedeon Richter and Læge Sofus Carl Emil Friis og Hustru Olga Doris Friis’ Fond. K.V. has received speakers’ fees from Gedeon Richter, Merck, and IBSA. L.N.V. has received grants, speakers’ fees, and conference fees (including travel support) from Merck Sharp & Dohme, and Ferring, and scientific board fees from Ferring. T.M.H. has received speakers’ fees from Merck, Merck Sharp & Dohme, and Ferring. A.P. has received speakers’ fees (including those classified as honoraria) from Ferring Pharmaceuticals, Merck, Gedeon Richter, IBSA, Abbott and Consulting fees from Gedeon Richter and Ferring and travel support from Gedeon Richter. H.S.N. received speakers’ fees from Ferring Pharmaceuticals, Merck, Astra Zeneca, Cook Medical, Gedeon Richter, Ibsa Nordic, Novo Nordisk, and Bessins. B.W.M. reports consulting fees, travel support, and research funding from Merck and consulting fees from Ferring, Organon, Repronovo, UNILAB, Vitra, and Norgine. N.l.C.F. has received speakers’ fees from Merck and Ferring Pharmaceuticals, consulting fees from Merck, and meeting support/registration fees from Merck, Ferring Pharmaceuticals, IBSA, and Gedeon Richter (paid to institution). She is also an unpaid chair in the steering committee for the guideline groups of The Danish Fertility Society. All other authors declare no competing interests

**REGISTRATION NUMBER:**

CRD42023479967.

## Introduction

Globally, the estimated lifetime prevalence of infertility is 17.5%, with a 12-month prevalence of 12.6% (Cox *et al*., 2022). To help these couples, the use of ART has expanded rapidly, with nearly 4 million ART cycles performed annually and ∼1 million ART-conceived babies born each year (Kupka *et al*., 2024). ICSI was originally introduced to overcome the limitations of conventional IVF (cIVF) in cases of severe male factor infertility (Palermo *et al*., 1992). Since its introduction, the use of ICSI has gradually grown beyond its original indication, despite an early randomized controlled trial (RCT) showing no benefit over cIVF (Bhattacharya *et al*., 2001). It has been widely assumed that ICSI might improve fertilization and thereby increase the likelihood of live birth while also reducing the risk of total fertilization failure. Consequently, many reproductive endocrinologists and patients prefer ICSI as the first-choice method, leading to its global use now exceeding that of cIVF (Wyns *et al*., 2020). Currently, ICSI accounts for nearly 60% of ART cycles worldwide, though with marked regional variation (Africa: 89%, Latin America: 88%, North America: 79%, Europe: 72%, Asia excluding China: 59%, Asia including China: 40% (Kupka *et al*., 2024), and Australia and New Zealand: 55% (Kotevski DP *et al*., 2025).

A Cochrane review published in 2023 concluded that available evidence did not demonstrate superiority of either method (ICSI or cIVF) in terms of live birth rates among couples with non-male factor infertility (Cutting *et al*., 2023). However, this conclusion was based on only two studies (Bhattacharya *et al*., 2001; Dang *et al*., 2021), one of which had a notably small sample size (Bhattacharya *et al*., 2001), and the authors highlighted the need for further investigation (Cutting *et al*., 2023). Since then, two additional large RCTs have been published (Wang *et al*., 2024; Berntsen *et al*., 2025), both using live birth as the main outcome measure. Together with the previous large RCT (Dang *et al*., 2021), these studies now provide a broader knowledge base. On this basis, we conducted a systematic review and meta-analysis of RCTs to evaluate the treatment outcomes of ICSI versus cIVF in couples without severe male factor infertility, focusing on live birth and cumulative live birth rates per couple.

## Materials and methods

### Information sources

This systematic review and meta-analysis was reported following the Preferred Reporting Items for Systematic Review and Meta-Analysis 2020 guidelines (PRIMSA 2020). The protocol was registered prior to data extraction at the International Prospective Register of Systematic Review registry (PROSPERO) (CRD42023479967).

### Search strategy

The search period ranged from the inception of the databases to 31 May 2025. Ten online academic databases (PubMed, EMBASE, MEDLINE, Web of Science, Cochrane Library, ProQuest Dissertations & Theses Global, Scopus, CINAHL Plus, Chinese Wan Fang Database, and Chinese CNKI Database) were searched without language restrictions. We performed a secondary search for gray literature in Google Scholar and WHO Library, as well as citation search by checking the reference lists of identified relevant studies after full-text screening. The detailed search strategy is provided in Supplementary Table S1.

### Eligibility criteria

Inclusion criteria were original RCTs comparing the outcomes of ICSI versus cIVF per couple, with at least one outcome compared between groups, having full-text access in the electronic library databases of Peking University, and written in English or Chinese. Although studies without accessible full texts after all retrieval efforts were excluded, any substantive study relevant to our research question would have been identified through other accessible citations.

### Outcomes

The primary outcome was the live birth rate, defined as the proportion of couples who achieved a live birth after the first embryo transfer. Critical secondary outcomes included the cumulative live birth rate and clinical pregnancy rate. Other secondary outcomes included the rates of fertilization, total fertilization failure, implantation, ongoing pregnancy, miscarriage, stillbirth, preterm birth, low birth weight, birth defect, neonatal death, multiple pregnancy, ectopic pregnancy, gestational diabetes, and gestational hypertension. Definitions of these secondary outcomes are provided in the Supplementary Information.

### Study selection and data extraction

Two independent investigators (K.K. and Y.F.) selected eligible articles based on titles, abstracts, and full texts. Data from included studies were extracted by one investigator (K.K.) and independently verified by another (Y.Y.). Due to the varying calculation formulas used for these outcomes in the included studies, all outcomes, except for fertilization and implantation rate, were recalculated using the number of randomized couples as the denominator. To ensure consistency across studies, unpublished data were obtained from the original investigators: cumulative live birth and ongoing pregnancy rate, and the fertilization rate from one study (Berntsen *et al*., 2025); fertilization rates of two studies (Dang *et al*., 2021; Wang *et al*., 2024). Clinical pregnancy and multiple pregnancy data (calculated per couple) from another trial (Bhattacharya *et al*., 2001) were extracted from a previously published review (Cutting *et al*., 2023).

### Trustworthiness and quality of studies

Studies that met trustworthiness criteria for data and results were identified by two independent investigators (K.K. and Y.W.) using the TRACT checklist (Mol *et al*., 2023). Only studies that fulfilled the predefined trustworthiness criteria were included. Study quality was evaluated by two independent investigators (K.K. and Y.F.) using the Cochrane Collaboration’s Risk of Bias 2 Tool (Sterne *et al*., 2019).

### Statistical analysis

The meta-analysis was performed using Revman 5.4.1, except for sensitivity analyses based on the restricted maximum likelihood (REML) estimator, which were conducted using meta package (version 8.2-0) in R. Random-effect models were applied to pool effect estimates with corresponding 95% CIs.

Risk ratios (RRs) were pooled for dichotomous variables, and mean differences (MDs) for continuous variables. Potential publication bias was assessed using funnel plots. Subgroup analyses were conducted according to the medical indications for ICSI. Two sensitivity analyses were performed for the primary outcome and critical secondary outcomes. First, studies that did not meet the predefined trustworthiness criteria were included to assess the robustness of the results. Second, we evaluated the sensitivity of the findings to the choice of heterogeneity estimator by having two investigators independently conduct the meta-analysis: K.K. used the DerSimonian–Laird (D-L) estimator, whereas D.F.S. used the REML estimator. GRADE decision-making was used to assess the quality of evidence (Balshem *et al*., 2011).

## Results

### Study selection

**Figure 1.**
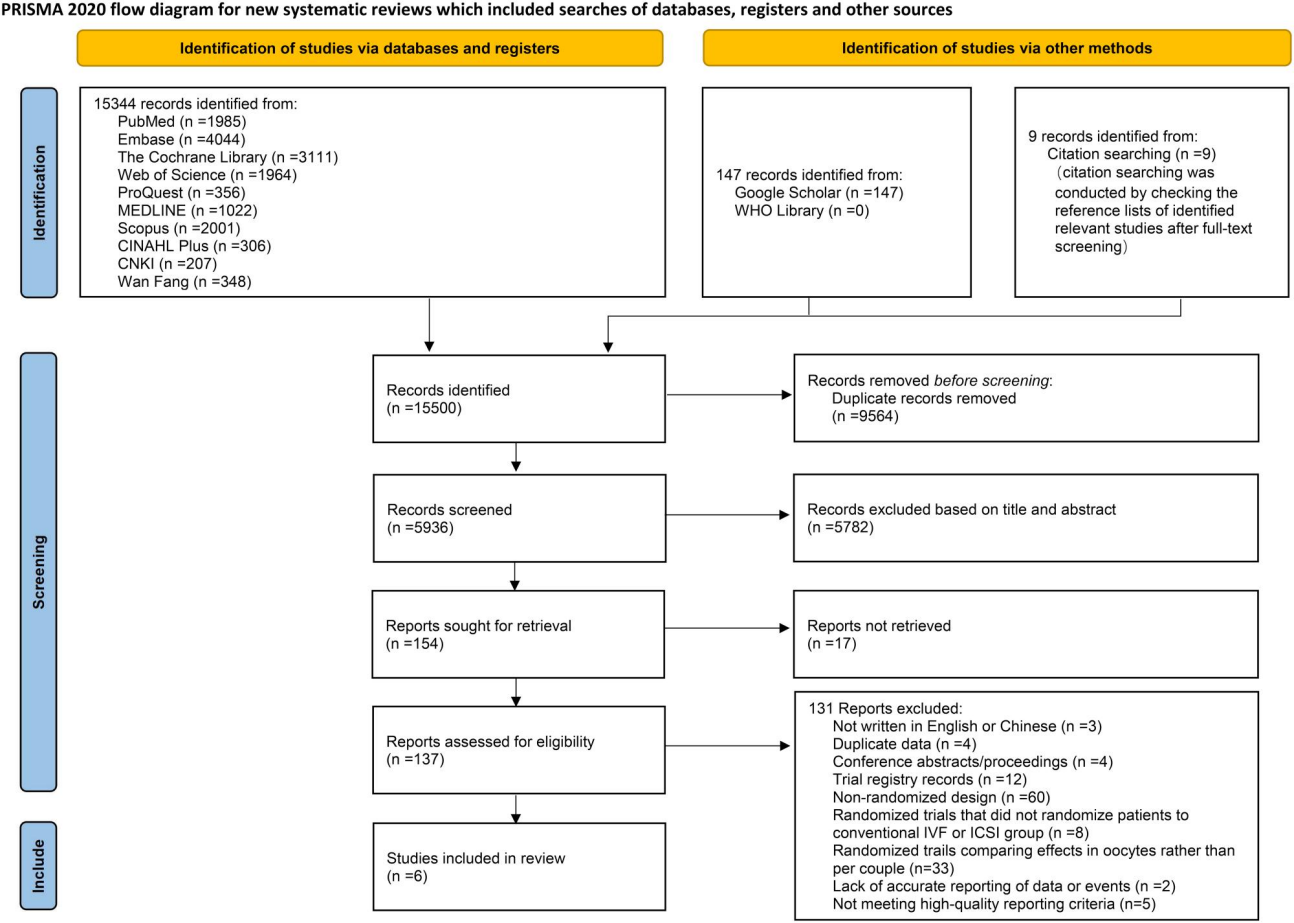
**PRISMA 2020 flowchart for the selection of studies.** Selection of studies comparing ART treatment outcomes between two fertilization methods (ICSI and cIVF). cIVF, conventional IVF.

The initial database search identified 15 344 records from PubMed (1985), Embase (4044), The Cochrane Library (3111), Web of Science (1964), ProQuest (356), MEDLINE (1022), Scopus (2001), CINAHL Plus (306), CNKI (207), and Wan Fang (348). An additional 147 records were identified through gray literature searched in Google Scholar (147) and the WHO Library (0), and nine more were identified by checking the reference lists of relevant studies after full-text screening. After removing duplicates and screening titles and abstracts, 154 records were selected for full-text review, of which 137 full-text articles were retrieved. Among these, 3 were not written in English or Chinese, 4 were duplicates, 4 were conference abstracts or proceedings, 12 were trial registry records, 60 used non-randomized designs, 8 were randomized trials that did not randomize participants to cIVF or ICSI, 33 compared effects per oocyte rather than per couple, 2 lacked accurate reporting of data or events, and 5 did not meet the predefined trustworthiness criteria. After exclusions, six RCTs were included in the main meta-analysis (Fig. 1). The five studies that did not meet the trustworthiness criteria were included in the subsequent sensitivity analysis.

### Study characteristics

Six RCTs (Bhattacharya *et al*., 2001; Foong *et al*., 2006; Dang *et al*., 2021; Fancsovits *et al*., 2023; Wang *et al*., 2024; Berntsen *et al*., 2025) met the inclusion criteria and were included in this meta-analysis. Sample sizes ranged from 60 to 2387 couples. Four trials (Bhattacharya *et al*., 2001; Dang *et al*., 2021; Wang *et al*., 2024; Berntsen *et al*., 2025) had multicenter designs. The reported indications for ART treatment included normal or non-severe male infertility (Wang *et al*., 2024; Berntsen *et al*., 2025), non-male factor infertility with unspecified type (e.g. tubal factor infertility, unexplained infertility, endometriosis, diminished ovarian reserve, or ovulation disorder) (Bhattacharya *et al*., 2001; Dang *et al*., 2021), tubal factor infertility (Aboulghar *et al*., 1996; Bukulmez *et al*., 2000; Dang *et al*., 2021), endometriosis (Dang *et al*., 2021), low oocyte number or advanced maternal age (Fancsovits *et al*., 2023), and unexplained infertility (Foong *et al*., 2006; Dang *et al*., 2021) (Table 1). None of the included studies involved couples with severe male factor infertility. Additional detailed information on the included studies is provided in Supplementary Tables S2 and S3, while definitions of the secondary outcomes are provided in Supplementary Table S4.

**Table 1.** Characteristics of the included studies.

Study ID	Country	Setting	Sample size	Duration of recruiting participants	Study population	Causes of infertility	Live birth per woman reported as an outcome
Bhattacharya *et al.* (2001)	UK	Multicenter	415 couples	Not reported	Non-male factor infertility	Non-male factor infertility with unspecified type	No
Foong *et al.* (2006)	Canada	Single center	60 couples	1997–2001	Non-male factor infertility	Unexplained infertility	Yes
Dang *et al.* (2021) *	Vietnam	Multicenter	1064 couples	2018–2019	Non-male factor infertility	Non-male factor infertility with unspecified type*	Yes
Fancsovits *et al.* (2023)	Hungary	Single center	336 cycles (randomized by couples)	2018–2020	Non-male factor infertility	Low oocyte number, advanced maternal age	No
Wang *et al.* (2024)	China	Multicenter	2387 couples	2018–2021	Male factor infertility	Non-severe male factor infertility	Yes
Berntsen *et al.* (2025)	Denmark	Multicenter	822 couples/women	2019–2022	Normal or male factor infertility	Normal or non-severe male factor infertility	Yes

* Subgroup analyses on specific indications including tubal factor infertility, unexplained infertility, and endometriosis were conducted in this study.

### Trustworthiness and quality of studies

Six studies (Bhattacharya *et al*., 2001; Foong *et al*., 2006; Dang *et al*., 2021; Fancsovits *et al*., 2023; Wang *et al*., 2024; Berntsen *et al*., 2025) met the predefined trustworthiness criteria, while another five studies (Sun *et al*., 2014; Wang *et al*., 2015; Sharula and Hatu 2016; Bi 2020; Xu *et al*., 2022) did not meet these criteria (Supplementary Table S5). The risk of bias assessments are presented in Supplementary Figs S1 and S2. Approximately two-thirds of the included studies were assessed as being at low risk of bias, while the remaining studies were judged to have some concerns. Funnel plots are shown in Supplementary Fig. S3. Egger’s test *P*-values were >0.05 for almost all outcomes.

### Outcomes in all included couples without severe male factor infertility

**Figure 2.**
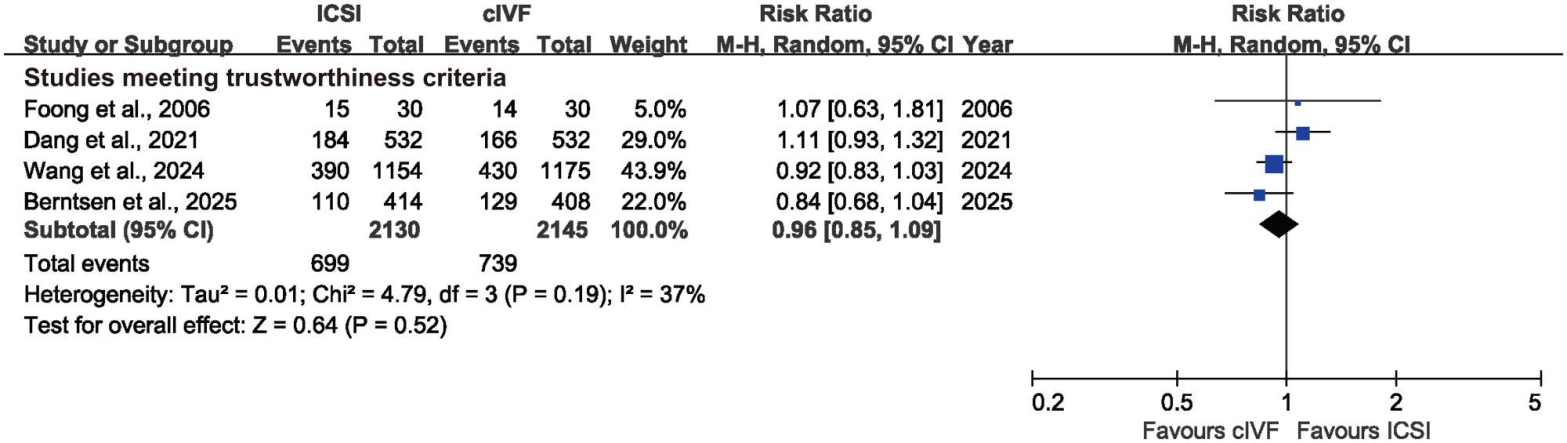
**Forest plot of livebirth rate in couples without severe male factor infertility.** cIVF, conventional IVF; RR, risk ratio.

**Figure 3.**
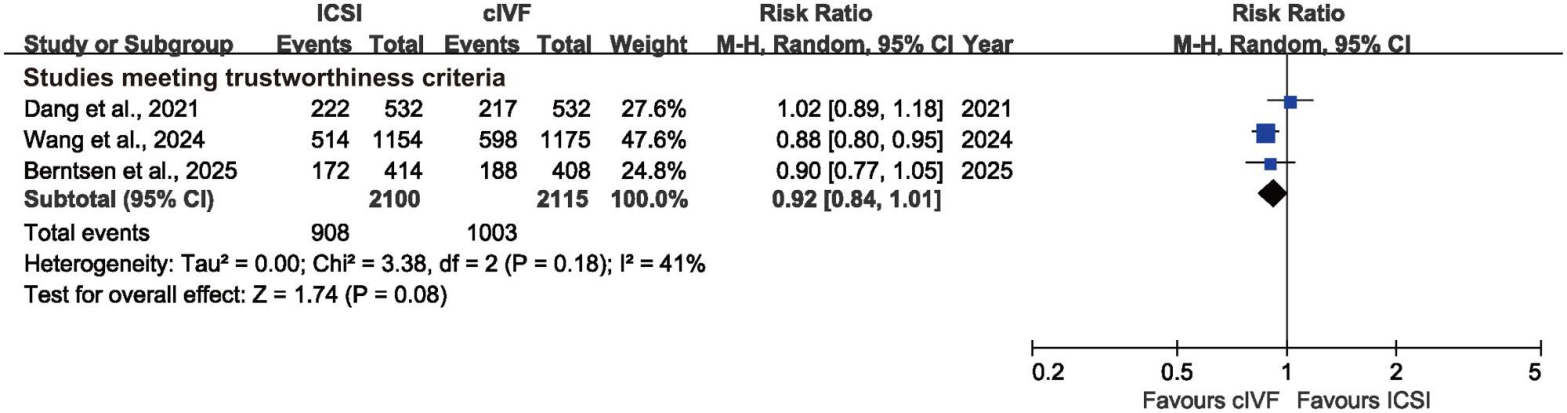
**Forest plot of cumulative livebirth rate in couples without severe male factor infertility.** cIVF, conventional IVF; RR, risk ratio.

**Figure 4.**
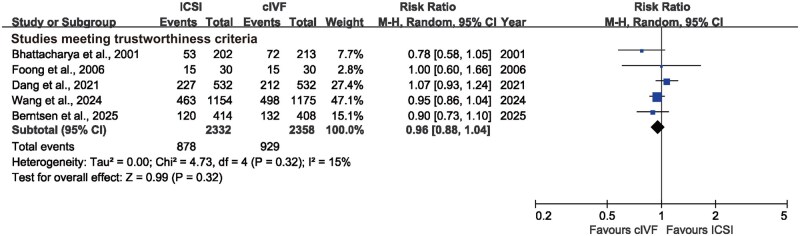
**Forest plot of clinical pregnancy rate in couples without severe male factor infertility.** cIVF, conventional IVF; RR, risk ratio.

**Figure 5.**
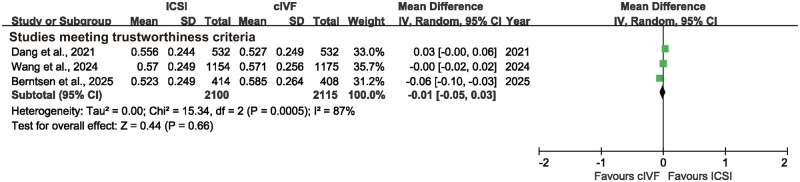
**Forest plot of fertilization rate in couples without severe male factor infertility.** cIVF, conventional IVF; MD, mean difference.

The meta-analysis showed no significant benefit of ICSI over cIVF in live birth rate (four studies, N = 1438, 32.8% vs 34.5%, pooled RR = 0.96, 95% CI: 0.85–1.09, *I*^2^ = 37%, high-quality evidence) (Fig. 2), cumulative live birth rate (three studies, N = 1911, 43.2% vs 47.4%, pooled RR = 0.92, 95% CI: 0.84–1.01, *I*^2^ = 41%, high-quality evidence) (Fig. 3), clinical pregnancy rate (five studies, N = 1807, 37.7% vs 39.4%, pooled RR = 0.96, 95% CI: 0.88–1.04, *I*^2^ = 15%, high-quality evidence) (Fig. 4), or fertilization rate (three studies, N = 4215, MD = −0.01, 95% CI: −0.01 to 0.03, *I*^2^ = 87%, moderate-quality evidence) (Fig. 5). Other infertility outcomes were comparable between groups; these included total fertilization failure rate (four studies, N = 198, 4.3% vs 5.0%, pooled RR = 0.86, 95% CI: 0.65–1.13, *I*^2^ = 0%, high-quality evidence) (Supplementary Fig. S4), implantation rate (six studies, N = 2336, 31.0% vs 33.4%, pooled RR = 0.93, 95% CI: 0.86–1.01, *I*^2^ = 17%, high-quality evidence) (Supplementary Fig. S5), ongoing pregnancy rate (three studies, N = 1451, 33.6% vs 35.2%, pooled RR = 0.96, 95% CI: 0.85–1.08, *I*^2^ = 43%, high-quality evidence) (Supplementary Fig. S6), and miscarriage rate (three studies, N = 201, 5.2% vs 4.3%, pooled RR = 1.19, 95% CI: 0.91–1.56, *I*^2^ = 0%, high-quality evidence) (Supplementary Fig. S7). There was a marginally significant difference in preterm birth rate between the two groups (three studies, N = 222, 4.6% vs 6.0%, pooled RR = 0.77, 95% CI: 0.59–1.00, *P* = 0.0447, *I*^2^ = 0%, high-quality evidence) (Supplementary Fig. S8). Other pregnancy outcomes were comparable between groups; these included stillbirth rate (one study, N = 1, 0.0% vs 0.2%, RR = 0.33, 95% CI: 0.01–8.04, high-quality evidence; data not shown), low birth weight rate (three studies, N = 128, 3.2% vs 2.9%, pooled RR = 1.10, 95% CI: 0.78–1.56, *I*^2^ = 0%, high-quality evidence) (Supplementary Fig. S9), birth defect rate (three studies, N = 39, 0.7% vs 1.1%, pooled RR = 0.63, 95% CI: 0.33–1.20, *I*^2^ = 0%, high-quality evidence) (Supplementary Fig. S10), neonatal death rate (one study, N = 3, 0.2% vs 0.1%, RR = 2.04, 95% CI: 0.18–22.43, moderate-quality evidence; data not shown), multiple pregnancy rate (three studies, N = 385, 7.7% vs 8.9%, pooled RR = 0.86, 95% CI: 0.71–1.04, *I*^2^ = 0%, high-quality evidence) (Supplementary Fig. S11), ectopic pregnancy rate (three studies, N = 51, 1.0% vs 1.3%, pooled RR = 0.83, 95% CI: 0.48–1.43, *I*^2^ = 0%, high-quality evidence) (Supplementary Fig. S12), gestational diabetes rate (three studies, N = 135, 3.1% vs 3.3%, pooled RR = 0.96, 95% CI: 0.69–1.34, *I*^2^ = 0%, high-quality evidence) (Supplementary Fig. S13), and gestational hypertension rate (three studies, N = 64, 1.3% vs 1.7%, pooled RR = 0.73, 95% CI: 0.45–1.20, *I*^2^ = 0%, high-quality evidence) (Supplementary Fig. S14).

### Outcomes according to the different study populations

#### Normal or non-severe male factor infertility

Two studies (Wang *et al*., 2024; Berntsen *et al*., 2025) including couples with normal or non-severe male factor infertility were analyzed. The meta-analysis showed that the ICSI group had a lower live birth rate (N = 1059, 31.9% vs 35.3%, pooled RR = 0.91, 95% CI: 0.82–1.00, *P* = 0.0421, *I*^2^ = 0%, high-quality evidence), lower cumulative live birth rate (N = 1472, 43.8% vs 49.7%, pooled RR = 0.88, 95% CI: 0.82–0.95, *I*^2^ = 0%, high-quality evidence), and lower implantation rate (N = 1475, 35.7% vs 38.9%, pooled RR = 0.92, 95% CI: 0.85–1.00, *P* = 0.0384, *I*^2^ = 0%, high-quality evidence). There was no significant between-group difference in total fertilization failure (N = 133, 4.0% vs 4.5%, pooled RR = 0.94, 95% CI: 0.56–1.57, *I*^2^ = 48%, high-quality evidence), fertilization rate (N = two studies, MD = −0.03, 95% CI: −0.09 to 0.03, *I*^2^ = 88%, moderate-quality evidence), ongoing pregnancy rate (N = 1087, 32.9% vs 36.1%, pooled RR = 0.91, 95% CI: 0.83–1.01, *I*^2^ = 0%, high-quality evidence), miscarriage rate (N = 146, 5.2% vs 4.0%, pooled RR = 1.29, 95% CI: 0.94–1.78, *I*^2^ = 0%, high-quality evidence), preterm birth rate (N = 161, 4.5% vs 5.7%, pooled RR = 0.78, 95% CI: 0.58–1.06, *I*^2^ = 0%, high-quality evidence), low birth weight rate (N = 112, 3.6% vs 3.5%, pooled RR = 1.05, 95% CI: 0.73–1.51, *I*^2^ = 0%, high-quality evidence), and birth defect rate (N = 34, 0.8% vs 1.3%, pooled RR = 0.62, 95% CI: 0.31–1.24, *I*^2^ = 0%, high-quality evidence). Other safety outcomes, including multiple pregnancy rate (N = 229, 6.6% vs 7.9%, pooled RR = 0.85, 95% CI: 0.66–1.08, *I*^2^ = 0%, high-quality evidence), ectopic pregnancy rate (N = 31, 0.8% vs 1.1%, pooled RR = 0.73, 95% CI: 0.36–1.48, *I*^2^ = 0%, high-quality evidence), gestational diabetes rate (N = 83, 2.6% vs 2.7%, pooled RR = 0.98, 95% CI: 0.64–1.50, *I*^2^ = 0%, high-quality evidence), and gestational hypertension rate (N = 62, 1.7% vs 2.3%, pooled RR = 0.73, 95% CI: 0.44–1.20, *I*^2^ = 0%, high-quality evidence) were also comparable between groups (Supplementary Fig. S15).

#### Non-male factor infertility with unspecified type

Two studies (Bhattacharya *et al*., 2001; Dang *et al*., 2021) included couples with non-male factor infertility of unspecified type; one of these (Bhattacharya *et al*., 2001) included 11% of couples with mild male factor infertility in each group. Only one study (Dang *et al*., 2021) reported live birth rates, showing no significant difference between groups (N = 350, 34.6% vs 31.2%, RR = 1.11, 95% CI: 0.93–1.32, moderate-quality evidence). Meta-analysis of the two studies revealed no significant difference in clinical pregnancy rate (N = 564, 38.1% vs 38.1%, pooled RR = 0.94, 95% CI: 0.69–1.28, *I*^2^ = 73%, very low-quality evidence), implantation rate (N = 729, 27.5% vs 29.3%, pooled RR = 0.88, 95% CI: 0.66–1.18, *I*^2^ = 74%, very low-quality evidence), or multiple pregnancy rate (N = 156, 9.9% vs 11.1%, pooled RR = 0.89, 95% CI: 0.66–1.20, *I*^2^ = 0%, low-quality evidence) between two groups (Supplementary Fig. S16).

#### Tubal factor infertility

One study (Dang *et al*., 2021) conducted a subgroup analysis in couples with tubal factor infertility, reporting no significant difference in the live birth rate between groups (N = 76, 30.2% vs 23.4%, pooled RR = 1.28, 95% CI: 0.89–1.83, high-quality evidence).

#### Endometriosis

One study (Dang *et al*., 2021) conducted a subgroup analysis in couples with endometriosis and found no difference in live birth rate between ICSI and cIVF (N = 11, 40.0% vs 18.8%, RR = 2.13, 95% CI: 0.67–6.76, low-quality evidence).

#### Low oocyte number

One study (Fancsovits *et al*., 2023) conducted a subgroup analysis in couples with low oocyte yield (≤4 oocytes collected) but did not report the live birth rates per couple. The implantation rates were comparable (N = 42, 7.2% vs 10.8%, RR = 0.67, 95% CI: 0.37–1.22, high-quality evidence).

#### Advanced maternal age

One study (Fancsovits *et al*., 2023) conducted a subgroup analysis in couples of advanced maternal age (≥40 years old) but did not report live birth rates per couple. There were no significant differences in the implantation rate (N = 40, 11.0% vs 16.0%, RR = 0.68, 95% CI: 0.38–1.24, high-quality evidence).

#### Unexplained infertility

Two studies (Foong *et al*., 2006; Dang *et al*., 2021) included couples with unexplained infertility. The meta-analysis showed no significant difference in live birth rate (N = 167, 38.4% vs 37.1%, pooled RR = 1.04, 95% CI: 0.82–1.32, *I*^2^ = 0%, moderate-quality evidence) (Supplementary Fig. S17). Only one study (Foong *et al*., 2006) reported additional outcomes, all of which were comparable between groups, including clinical pregnancy (N = 30, 50.0% vs 50.0%, RR = 0.87, 95% CI: 0.50–1.49, moderate-quality of evidence), total fertilization failure (N = 2, 0% vs 6.7%, RR = 0.20, 95% CI: 0.01–4.00, low-quality of evidence), and implantation (N = 63, 44.4% vs 38.2%, RR = 1.16, 95% CI: 0.80–1.70, moderate-quality of evidence).

### Quality of evidence

Summary of findings tables were prepared for all outcomes in the main analyses and subgroup analyses, following the GRADE approach (Supplementary Table S6).

### Sensitivity analysis

Sensitivity analyses, including studies that did not meet the trustworthiness criteria, yielded similar results, with wider 95% CIs (Supplementary Figs S18 and S19). Sensitivity analyses using the REML estimator instead of the D-L method produced comparable effect estimates (Supplementary Figs S20, S21, and S22).

## Discussion

In this systematic review and meta-analysis of six RCTs (n = 5084) comparing ICSI with cIVF in couples without severe male factor infertility, we found high-certainty evidence that ICSI was not associated with higher live birth rates or the cumulative live birth rates. However, ICSI was associated with a borderline reduction in preterm birth rate (<37 weeks of gestation), while no significant differences were observed for other fertility or pregnancy outcomes. Subgroup analyses also showed that the available evidence did not support any advantage of ICSI over cIVF across a range of clinical indications, including non-severe male infertility, non-male factor infertility of unspecified type, tubal factor infertility, endometriosis, low oocyte number, advanced maternal age, and unexplained infertility. Although in the subgroup of couples with normal or non-severe male factor infertility, ICSI resulted in a lower live birth rate and cumulative live birth rate, these subgroup findings should be considered exploratory.

A single RCT rarely changes clinical guidelines, despite being the gold standard for evaluating treatments and interventions. Importantly, RCTs are not immune to bias. Such limitations can be mitigated by conducting multicenter RCTs, with even greater methodological robustness achieved through international, multi-setting trials led by independent investigators using diverse protocols, such as the large RCTs included in our analyses (Dang *et al*., 2021; Wang *et al*., 2024; Berntsen *et al*., 2025). In addition, the safety and efficacy of a new intervention are best established when multiple high-quality RCTs yield consistent findings (Gale *et al*., 2023). Meta-analyses, such as the present study, play a critical role in consolidating evidence, thereby forming a solid foundation for clinical guidelines and recommendations.

This is the first systematic review and meta-analysis to incorporate large datasets from several RCTs conducted across diverse international settings, thereby providing a more comprehensive and generalizable evidence base. To ensure the validity of randomization in the included studies, RCTs that randomized per oocyte were excluded. Additionally, we used the TRACT checklist to identify the high-quality RCTs that met trustworthiness criteria. The results of sensitivity analyses, which included studies that did not meet the trustworthiness criteria, support the robustness of the primary meta-analysis results and validate the decision to include only studies meeting the trustworthiness criteria in the main analysis. Together, these methodological safeguards strengthen the validity of our findings and support more reliable, evidence-based recommendations for clinical practice regarding the use of ICSI.

This study also has several limitations. First, only four studies reported live birth rates, while the others reported intermediate outcomes such as fertilization and pregnancy, which do not directly reflect the contribution to achieving a live birth. Second, because only a few small studies addressed single, specific treatment indications, it was not possible to draw definitive conclusions regarding the comparative effectiveness of the two methods in subgroup analyses. Third, although the subgroup comparisons focused on the specific indications listed in the subheadings, the study populations also included couples with a range of other infertility diagnoses; therefore, the subgroup results should be interpreted with caution. Additionally, heterogeneity was considerable for certain outcomes (Fig. 5; Supplementary Figs S15d and S16). This may partly reflect the highly operator-sensitive nature of the ICSI procedure itself. Differences in embryologist skill, experience, and technical execution between centers could influence laboratory outcomes such as fertilization rates. Future studies should therefore report more detailed laboratory quality control data, such as operator experience and the incidence of 0PN, 1PN, and 3PN embryos after ICSI, to allow future meta-analyses to better explore the sources of heterogeneity. Fourth, the presence of some concerns in some included studies introduces a degree of uncertainty regarding the risk of bias. Moreover, because all outcomes included fewer than 10 studies, neither visual inspection of funnel plots nor Egger’s test can reliably detect publication bias. Nevertheless, based on qualitative assessment, including prospective trial registration, publication in high-impact journals, and reporting of prespecified outcomes, the risk of publication bias appears low, although the presence of unreported studies cannot be entirely excluded. While outcome calculations were predefined, the definition of some outcomes remained inconsistent between studies due to variations in measurement strategies across countries. We accounted for heterogeneity between studies for all outcomes when pooling the estimates. Furthermore, to improve consistency, CLBR was limited to events occurring within 12 months after randomization, which could be extracted from several studies. Finally, in per-couple randomized trials, fertilization rate and implantation rate are not statistically independent. Although per-woman rates would be preferable, these could not be consistently derived because of heterogeneous reporting across studies within an aggregate-data meta-analytic framework.

Current guidelines on ICSI indications remain inconsistent. The European Society of Human Reproduction and Embryology (ESHRE) advises against ICSI over cIVF for non-male factor (Lundin *et al*., 2023) or unexplained infertility (Guideline Group on Unexplained Infertility *et al.*, 2023). The American Society for Reproductive Medicine (ASRM) similarly discourages routine ICSI use without male factor infertility or prior fertilization failure (ASRM 2020). The American Urological Association (AUA/ASRM) suggests ICSI may overcome poor sperm parameters if viable sperm are available (Schlegel *et al*., 2021a,b). The Chinese Association of Reproductive Medicine recommends ICSI for severe oligozoospermia, asthenozoospermia, specific teratozoospermia types, surgical sperm retrieval, sperm-related oocyte activation issues, antisperm antibodies, and selected non-male factors (e.g. Preimplantation Genetic Testing [PGT], in vitro maturation [IVM], frozen–thawed oocytes, prior cIVF failure, or zona abnormalities) (Li *et al*., 2023). Such variation in international recommendations continues to contribute to clinical uncertainty.

Historically, the preference for ICSI has been based on the unconfirmed hypothesis that it could lead to higher fertilization rates, thereby increasing the chances of achieving a live birth. However, our meta-analysis found that among couples without severe male factor infertility, ICSI was associated with a non-significant lower fertilization rate and implantation rate compared to cIVF. We also observed a marginally significant reduction in preterm birth <37 weeks in the ICSI group compared to the cIVF group (4.6% vs 6.0%, pooled RR = 0.77, 95% CI: 0.59–1.00). This difference is likely attributable to the higher incidence of multiple pregnancies in the cIVF group (Goldenberg *et al*., 2008), despite the comparable rates of double embryo transfer between the two groups.

Since ICSI is an invasive procedure that bypasses the natural selection process of sperms and oocytes, concerns about the safety of offspring conceived through ICSI have persisted over the past decades (Ludwig *et al*., 2001; Catford *et al*., 2017; Pereira *et al*., 2017; Inoue *et al*., 2019). Our analysis showed a comparable rate of birth defect between the two groups (0.7% vs 1.1%, pooled RR = 0.63, 95% CI: 0.33–1.20). However, this outcome was reported in only three trials (Dang *et al*., 2021; Wang *et al*., 2024; Berntsen *et al*., 2025), none of which were adequately powered or specifically designed to detect differences in birth defects. Therefore, further RCTs with larger sample sizes and prespecified safety outcomes are required to reliably assess these outcomes. Meanwhile, data from large observational studies (Davies *et al*., 2012; Henningsen *et al*., 2023; Zhang *et al*., 2024) suggest that ICSI may be associated with an increased risk of any birth defect compared with cIVF. Given the potential health risks of ICSI to offspring and its high treatment costs, caution should be exercised in the routine clinical use of ICSI. In fact, based on the current evidence, ICSI should be reserved for severe male factor infertility.

In conclusion, the available high-certainty evidence does not support the routine use of ICSI in couples without severe male factor infertility. Given the higher treatment costs and the potential health risks to offspring associated with ICSI, its use should be restricted to its original indication: severe male factor infertility. Future research should focus on well-defined patient subgroups, refinement of semen reference standards, and evaluation of emerging laboratory and sperm parameters that may better identify patients who could benefit from ICSI. In addition, continued systematic evaluations of long-term offspring outcomes are essential to inform clinical practice and guideline development.

## Supplementary Material

deag066_Supplementary_Figure_S1

deag066_Supplementary_Figure_S2

deag066_Supplementary_Figure_S3

deag066_Supplementary_Figure_S4

deag066_Supplementary_Figure_S5

deag066_Supplementary_Figure_S6

deag066_Supplementary_Figure_S7

deag066_Supplementary_Figure_S8

deag066_Supplementary_Figure_S9

deag066_Supplementary_Figure_S10

deag066_Supplementary_Figure_S11

deag066_Supplementary_Figure_S12

deag066_Supplementary_Figure_S13

deag066_Supplementary_Figure_S14

deag066_Supplementary_Figure_S15

deag066_Supplementary_Figure_S16

deag066_Supplementary_Figure_S17

deag066_Supplementary_Figure_S18

deag066_Supplementary_Figure_S19

deag066_Supplementary_Figure_S20

deag066_Supplementary_Figure_S21

deag066_Supplementary_Figure_S22

deag066_Supplementary_Table_S1

deag066_Supplementary_Table_S2

deag066_Supplementary_Table_S3

deag066_Supplementary_Table_S4

deag066_Supplementary_Table_S5

deag066_Supplementary_Table_S6

## Data Availability

The data underlying this article are available in the article and in its online supplementary material.
