# Supplementary figures and images for: Does intracytoplasmic sperm injection outperform conventional in vitro fertilization in couples without severe male factor infertility? A systematic review and meta-analysis of randomized controlled trials
Source: Hum Reprod. 2026 May 22;41(7):1173–82. doi: 10.1093/humrep/deag066 (PMC13334920; doi:10.1093/humrep/deag066)

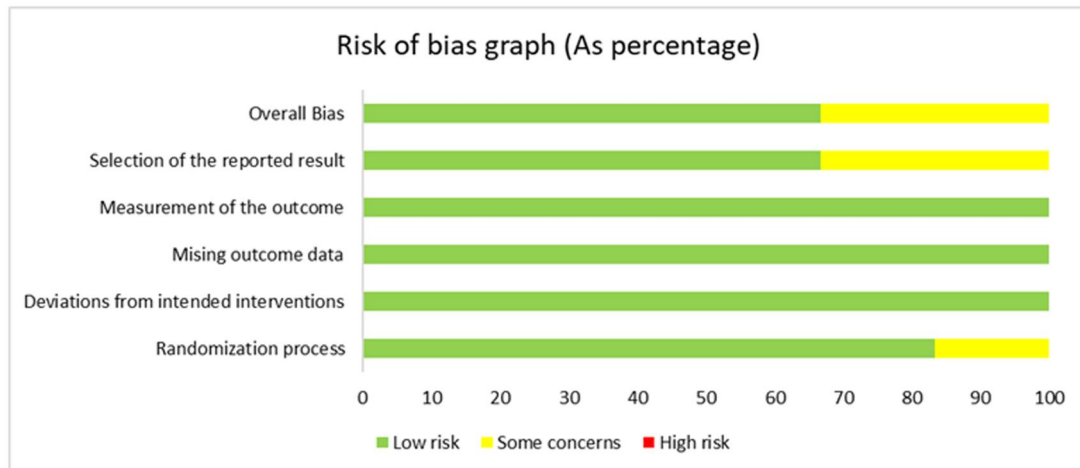

**Supplementary Figure S1.** Risk of bias graph.

Supplement: deag066_Supplementary_Figure_S1 [file deag066_supplementary_figure_s1.pdf]

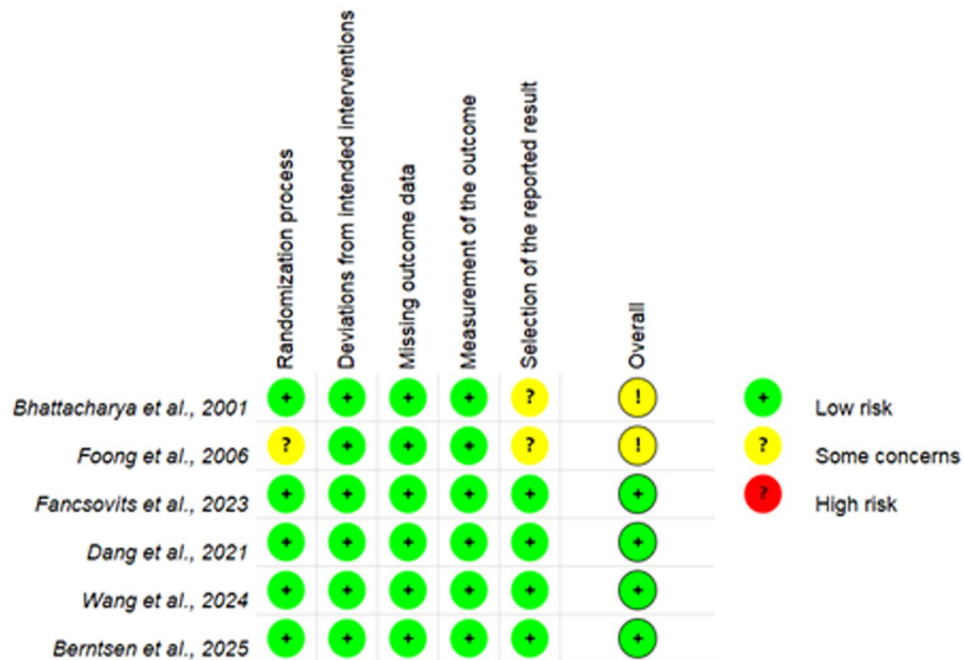

**Supplementary Figure S2.** Risk of bias summary.

Supplement: deag066_Supplementary_Figure_S2 [file deag066_supplementary_figure_s2.pdf]
