# Supplementary material for: Does intracytoplasmic sperm injection outperform conventional in vitro fertilization in couples without severe male factor infertility? A systematic review and meta-analysis of randomized controlled trials
Source: Hum Reprod. 2026 May 22;41(7):1173–82. doi: 10.1093/humrep/deag066 (PMC13334920; doi:10.1093/humrep/deag066)
Supplement: deag066_Supplementary_Figure_S3 [file deag066_supplementary_figure_s3.pdf]

Human Reproduction, 2026, 1–1
https://doi.org/10.1093/humrep/deag066
Supplementary Data

human reproduction
OXFORD

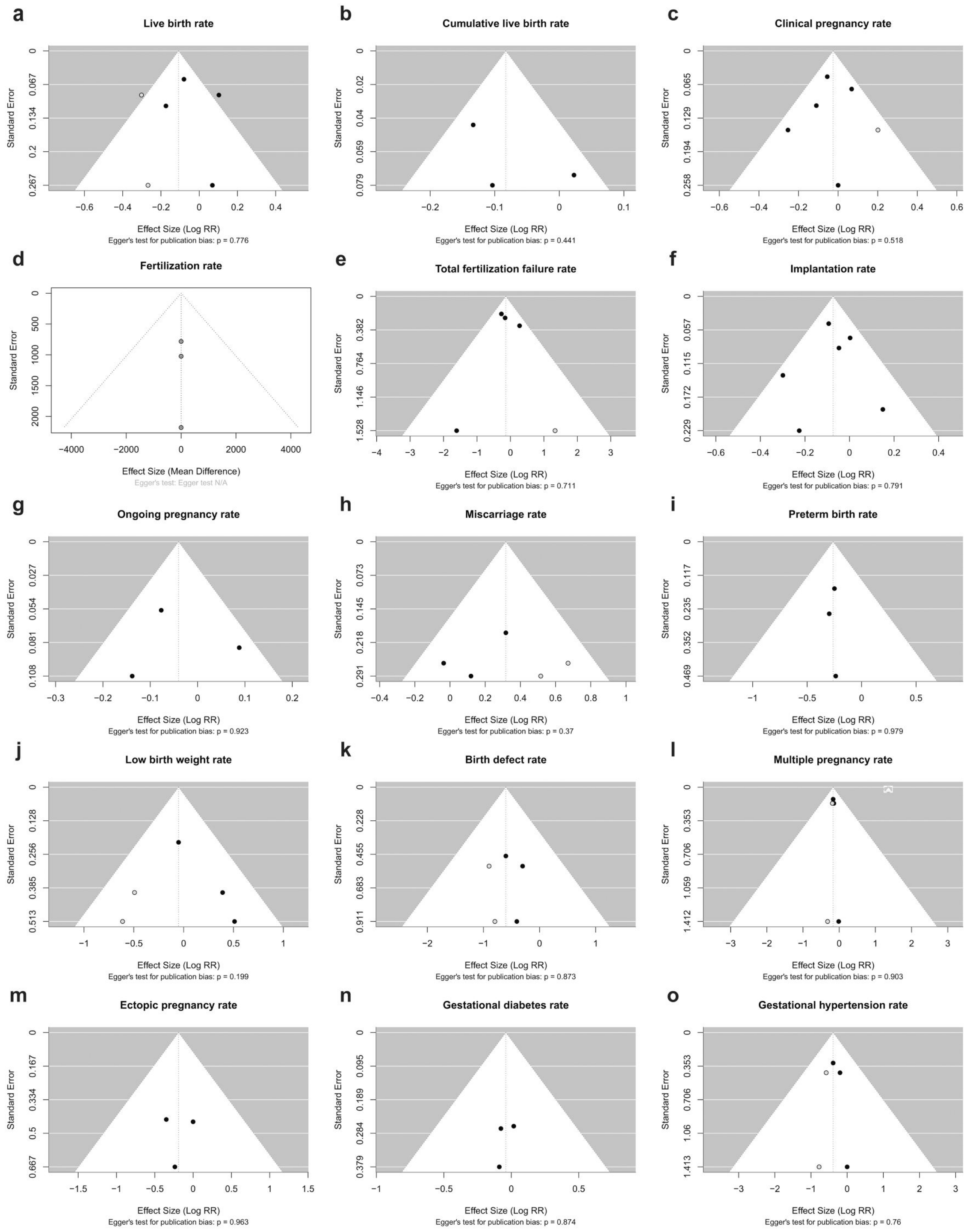

Supplementary Figure S3. Funnel plots in couples without severe male factor infertility. (**a**) Live birth rate; (**b**) cumulative live birth rate; (**c**) clinical pregnancy rate; (**d**) fertilization rate; (**e**) total fertilization failure rate; (**f**) implantation rate; (**g**) ongoing pregnancy rate; (**h**) miscarriage rate; (**i**) preterm birth rate; (**j**) low birth weight rate; (**k**) birth defect rate; (**l**) multiple pregnancy rate; (**m**) ectopic pregnancy rate; (**n**) gestational diabetes rate; (**o**) gestational hypertension rate. RR, risk ratio.
