# Supplementary material for: Does intracytoplasmic sperm injection outperform conventional in vitro fertilization in couples without severe male factor infertility? A systematic review and meta-analysis of randomized controlled trials
Source: Hum Reprod. 2026 May 22;41(7):1173–82. doi: 10.1093/humrep/deag066 (PMC13334920; doi:10.1093/humrep/deag066)
Supplement: deag066_Supplementary_Figure_S4 [file deag066_supplementary_figure_s4.pdf]

Human Reproduction, 2026, 1–1
https://doi.org/10.1093/humrep/deag066
Supplementary Data

human reproduction
OXFORD

	ICSI		cIVF			Risk Ratio	
Study or Subgroup	Events	Total	Events	Total	Weight	M-H, Random, 95% CI	Year
**Studies meeting trustworthiness criteria**							
Foong et al., 2006	0	30	2	30	0.8%	0.20 [0.01, 4.00]	2006
Dang et al., 2021	29	532	34	532	32.6%	0.85 [0.53, 1.38]	2021
Wang et al., 2024	42	1154	56	1175	49.0%	0.76 [0.52, 1.13]	2024
Berntsen et al., 2025	20	414	15	408	17.5%	1.31 [0.68, 2.53]	2025
**Subtotal (95% CI)**		**2130**		**2145**	**100.0%**	**0.86 [0.65, 1.13]**	
Total events	91		107				
Heterogeneity: Tau² = 0.00; Chi² = 2.87, df = 3 (P = 0.41); I² = 0%							
Test for overall effect: Z = 1.07 (P = 0.28)							

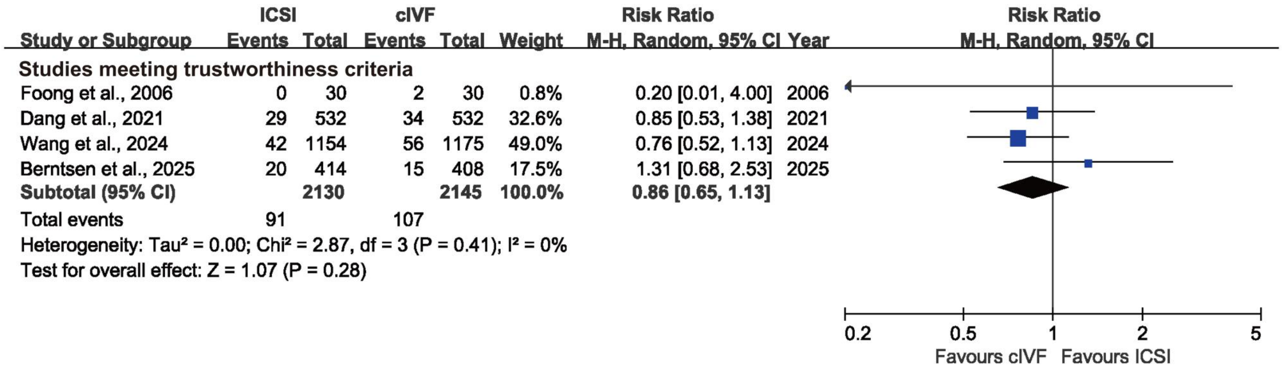

Supplementary Figure S4. **Forest plot of total fertilization failure rate in couples without severe male factor infertility.** cIVF, conventional IVF.
