# Supplementary material for: Does intracytoplasmic sperm injection outperform conventional in vitro fertilization in couples without severe male factor infertility? A systematic review and meta-analysis of randomized controlled trials
Source: Hum Reprod. 2026 May 22;41(7):1173–82. doi: 10.1093/humrep/deag066 (PMC13334920; doi:10.1093/humrep/deag066)
Supplement: deag066_Supplementary_Figure_S5 [file deag066_supplementary_figure_s5.pdf]

Human Reproduction, 2026, 1–1
https://doi.org/10.1093/humrep/deag066
Supplementary Data

human reproduction

OXFORD

Study or Subgroup	ICSI Events	ICSI Total	cIVF Events	cIVF Total	Weight	Risk Ratio M-H, Random, 95% CI	Year
**Studies meeting trustworthiness criteria**							
Bhattacharya et al., 2001	72	325	95	318	8.3%	0.74 [0.57, 0.97]	2001
Foong et al., 2006	32	72	31	81	4.2%	1.16 [0.80, 1.70]	2006
Dang et al., 2021	284	971	278	953	24.5%	1.00 [0.87, 1.15]	2021
Fancsovits et al., 2023	29	278	40	306	3.0%	0.80 [0.51, 1.25]	2023
Wang et al., 2024	564	1642	620	1644	42.6%	0.91 [0.83, 1.00]	2024
Berntsen et al., 2025	139	327	152	341	17.4%	0.95 [0.80, 1.13]	2025
**Subtotal (95% CI)**		**3615**		**3643**	**100.0%**	**0.93 [0.86, 1.01]**	
Total events	1120		1216				
Heterogeneity: Tau² = 0.00; Chi² = 6.00, df = 5 (P = 0.31); I² = 17%							
Test for overall effect: Z = 1.79 (P = 0.07)							

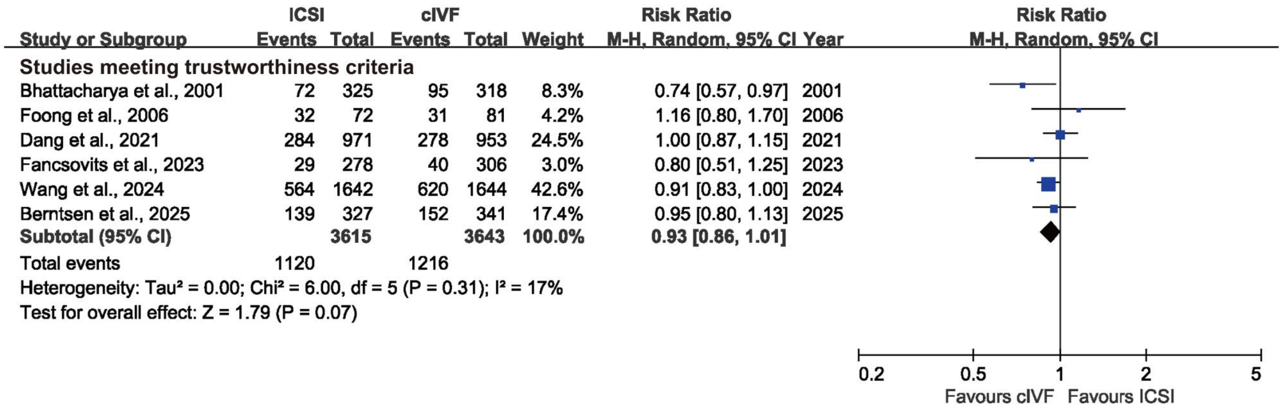

Supplementary Figure S5. Forest plot of implantation rate in couples without severe male factor infertility. cIVF, conventional IVF; RR, risk ratio.
