# Supplementary material for: Does intracytoplasmic sperm injection outperform conventional in vitro fertilization in couples without severe male factor infertility? A systematic review and meta-analysis of randomized controlled trials
Source: Hum Reprod. 2026 May 22;41(7):1173–82. doi: 10.1093/humrep/deag066 (PMC13334920; doi:10.1093/humrep/deag066)
Supplement: deag066_Supplementary_Figure_S6 [file deag066_supplementary_figure_s6.pdf]

Human Reproduction, 2026, 1–1
https://doi.org/10.1093/humrep/deag066
Supplementary Data

human reproduction
OXFORD

	ICSI		cIVF			Risk Ratio	
**Study or Subgroup**	**Events**	**Total**	**Events**	**Total**	**Weight**	**M-H, Random, 95% CI**	**Year**
**Studies meeting trustworthiness criteria**							
Dang et al., 2021	190	532	174	532	30.6%	1.09 [0.92, 1.29]	2021
Wang et al., 2024	402	1154	442	1175	46.9%	0.93 [0.83, 1.03]	2024
Berntsen et al., 2025	114	414	129	408	22.5%	0.87 [0.70, 1.08]	2025
**Subtotal (95% CI)**		**2100**		**2115**	**100.0%**	**0.96 [0.85, 1.08]**	
Total events	706		745				
Heterogeneity: Tau² = 0.00; Chi² = 3.52, df = 2 (P = 0.17); I² = 43%							
Test for overall effect: Z = 0.66 (P = 0.51)							

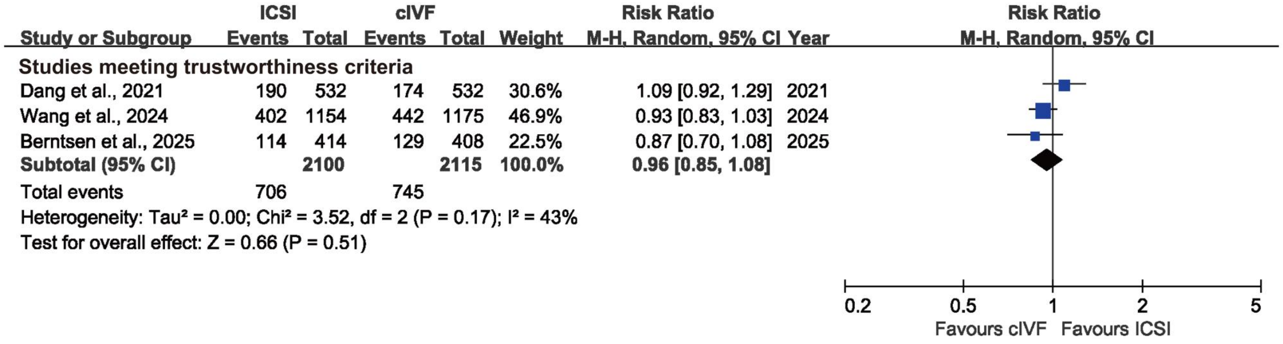

Supplementary Figure S6. Forest plot of ongoing pregnancy rate in couples without severe male factor infertility. cIVF, conventional IVF.
