# Supplementary material for: Does intracytoplasmic sperm injection outperform conventional in vitro fertilization in couples without severe male factor infertility? A systematic review and meta-analysis of randomized controlled trials
Source: Hum Reprod. 2026 May 22;41(7):1173–82. doi: 10.1093/humrep/deag066 (PMC13334920; doi:10.1093/humrep/deag066)
Supplement: deag066_Supplementary_Figure_S7 [file deag066_supplementary_figure_s7.pdf]

Human Reproduction, 2026, 1–1
https://doi.org/10.1093/humrep/deag066
Supplementary Data

human reproduction
OXFORD

Study or Subgroup	ICSI Events	ICSI Total	cIVF Events	cIVF Total	Weight	Risk Ratio M-H, Random, 95% CI	Year
**Studies meeting trustworthiness criteria**							
Dang et al., 2021	27	532	28	532	27.8%	0.96 [0.58, 1.61]	2021
Wang et al., 2024	58	1154	43	1175	49.5%	1.37 [0.93, 2.02]	2024
Berntsen et al., 2025	24	414	21	408	22.7%	1.13 [0.64, 1.99]	2025
**Subtotal (95% CI)**		**2100**		**2115**	**100.0%**	**1.19 [0.91, 1.56]**	
Total events	109		92				
Heterogeneity: Tau² = 0.00; Chi² = 1.21, df = 2 (P = 0.55); I² = 0%							
Test for overall effect: Z = 1.26 (P = 0.21)							

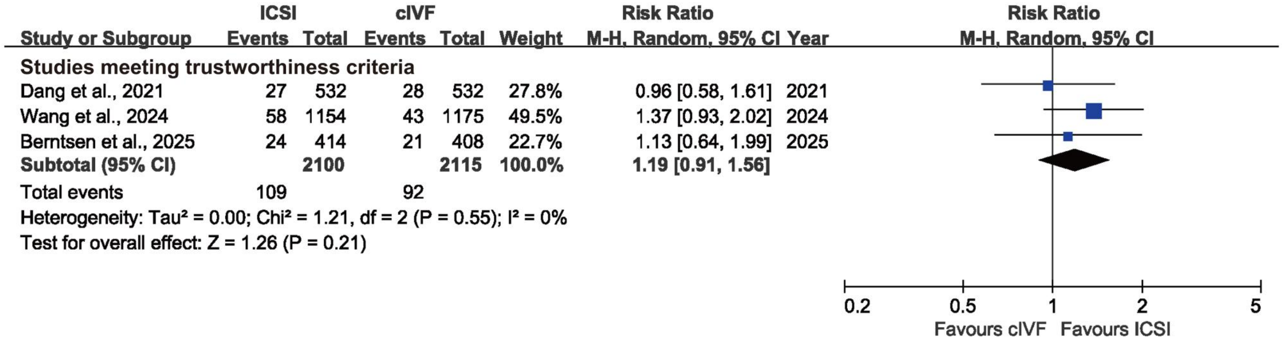

Supplementary Figure S7. Forest plot of miscarriage rate in couples without severe male factor infertility. cIVF, conventional IVF.
