# Supplementary material for: Does intracytoplasmic sperm injection outperform conventional in vitro fertilization in couples without severe male factor infertility? A systematic review and meta-analysis of randomized controlled trials
Source: Hum Reprod. 2026 May 22;41(7):1173–82. doi: 10.1093/humrep/deag066 (PMC13334920; doi:10.1093/humrep/deag066)
Supplement: deag066_Supplementary_Figure_S8 [file deag066_supplementary_figure_s8.pdf]

Human Reproduction, 2026, 1–1
https://doi.org/10.1093/humrep/deag066
Supplementary Data

	ICSI		cIVF			Risk Ratio	
Study or Subgroup	Events	Total	Events	Total	Weight	M-H, Random, 95% CI	Year
**Studies meeting trustworthiness criteria**							
Dang et al., 2021	26	532	35	532	27.4%	0.74 [0.45, 1.22]	2021
Wang et al., 2024	62	1154	81	1175	64.7%	0.78 [0.57, 1.07]	2024
Berntsen et al., 2025	8	414	10	408	7.9%	0.79 [0.31, 1.98]	2025
**Subtotal (95% CI)**		**2100**		**2115**	**100.0%**	**0.77 [0.59, 1.00]**	
Total events	96		126				
Heterogeneity: Tau² = 0.00; Chi² = 0.03, df = 2 (P = 0.99); I² = 0%							
Test for overall effect: Z = 1.99 (P = 0.05)							

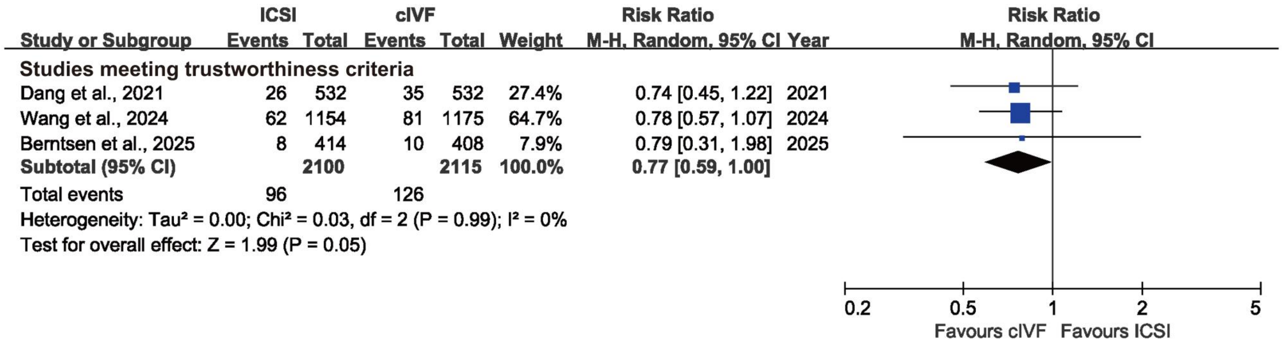

Supplementary Figure S8. Forest plot of preterm birth rate <37 weeks in couples without severe male factor infertility. cIVF, conventional IVF.
