# Supplementary material for: Does intracytoplasmic sperm injection outperform conventional in vitro fertilization in couples without severe male factor infertility? A systematic review and meta-analysis of randomized controlled trials
Source: Hum Reprod. 2026 May 22;41(7):1173–82. doi: 10.1093/humrep/deag066 (PMC13334920; doi:10.1093/humrep/deag066)
Supplement: deag066_Supplementary_Figure_S9 [file deag066_supplementary_figure_s9.pdf]

*Human Reproduction*, 2026, 1–1
https://doi.org/10.1093/humrep/deag066
**Supplementary Data**

human reproduction
OXFORD

| Study or Subgroup | ICSI Events | ICSI Total | cIVF Events | cIVF Total | Weight | Risk Ratio M-H, Random, 95% CI | Year |
|---|---|---|---|---|---|---|---|
| **Studies meeting trustworthiness criteria** | | | | | | | |
| Dang et al., 2021 | 10 | 532 | 6 | 532 | 11.7% | 1.67 [0.61, 4.55] | 2021 |
| Wang et al., 2024 | 42 | 1154 | 45 | 1175 | 69.3% | 0.95 [0.63, 1.44] | 2024 |
| Berntsen et al., 2025 | 15 | 414 | 10 | 408 | 19.0% | 1.48 [0.67, 3.25] | 2025 |
| **Subtotal (95% CI)** | | **2100** | | **2115** | **100.0%** | **1.10 [0.78, 1.56]** | |
| Total events | 67 | | 61 | | | | |
| Heterogeneity: Tau² = 0.00; Chi² = 1.68, df = 2 (P = 0.43); I² = 0% | | | | | | | |
| Test for overall effect: Z = 0.56 (P = 0.57) | | | | | | | |

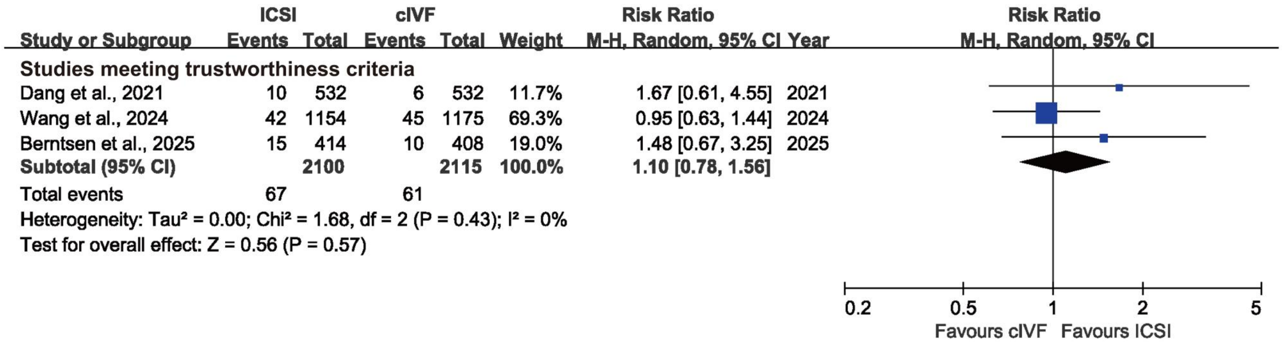


**Supplementary Figure S9. Forest plot of low birth weight rate in couples without severe male factor infertility.** cIVF, conventional IVF.
