# Supplementary material for: Does intracytoplasmic sperm injection outperform conventional in vitro fertilization in couples without severe male factor infertility? A systematic review and meta-analysis of randomized controlled trials
Source: Hum Reprod. 2026 May 22;41(7):1173–82. doi: 10.1093/humrep/deag066 (PMC13334920; doi:10.1093/humrep/deag066)
Supplement: deag066_Supplementary_Figure_S12 [file deag066_supplementary_figure_s12.pdf]

Human Reproduction, 2026, 1–1
https://doi.org/10.1093/humrep/deag066
Supplementary Data

human reproduction
OXFORD

Study or Subgroup	ICSI Events	ICSI Total	cIVF Events	cIVF Total	Weight	Risk Ratio M-H, Random, 95% CI	Year
**Studies meeting trustworthiness criteria**							
Dang et al., 2021	10	532	10	532	40.1%	1.00 [0.42, 2.38]	2021
Wang et al., 2024	9	1154	13	1175	42.2%	0.70 [0.30, 1.64]	2024
Berntsen et al., 2025	4	414	5	408	17.7%	0.79 [0.21, 2.92]	2025
**Subtotal (95% CI)**		**2100**		**2115**	**100.0%**	**0.83 [0.48, 1.43]**	
Total events	23		28				
Heterogeneity: Tau² = 0.00; Chi² = 0.33, df = 2 (P = 0.85); I² = 0%							
Test for overall effect: Z = 0.68 (P = 0.50)							

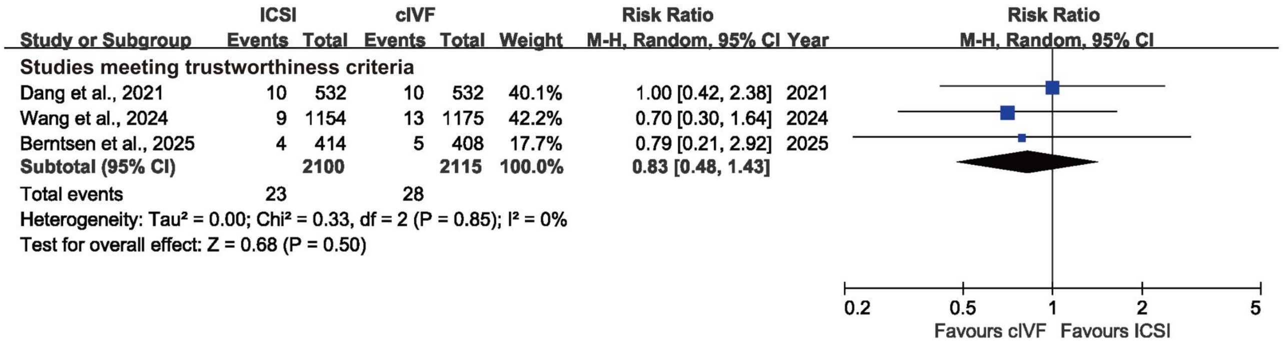

Supplementary Figure S12. **Forest plot of ectopic pregnancy rate in couples without severe male factor infertility.** cIVF, conventional IVF.
