# Supplementary material for: Does intracytoplasmic sperm injection outperform conventional in vitro fertilization in couples without severe male factor infertility? A systematic review and meta-analysis of randomized controlled trials
Source: Hum Reprod. 2026 May 22;41(7):1173–82. doi: 10.1093/humrep/deag066 (PMC13334920; doi:10.1093/humrep/deag066)
Supplement: deag066_Supplementary_Figure_S14 [file deag066_supplementary_figure_s14.pdf]

Human Reproduction, 2026, 1–1
https://doi.org/10.1093/humrep/deag066
Supplementary Data

human reproduction

OXFORD

Study or Subgroup	ICSI Events	ICSI Total	cIVF Events	cIVF Total	Weight	Risk Ratio M-H, Random, 95% CI	Year
**Studies meeting trustworthiness criteria**							
Dang et al., 2021	1	532	1	532	3.2%	1.00 [0.06, 15.95]	2021
Wang et al., 2024	16	1154	24	1175	61.5%	0.68 [0.36, 1.27]	2024
Berntsen et al., 2025	10	414	12	408	35.3%	0.82 [0.36, 1.88]	2025
**Subtotal (95% CI)**		**2100**		**2115**	**100.0%**	**0.73 [0.45, 1.20]**	
Total events	27		37				

Heterogeneity: Tau² = 0.00; Chi² = 0.18, df = 2 (P = 0.91); I² = 0%

Test for overall effect: Z = 1.23 (P = 0.22)

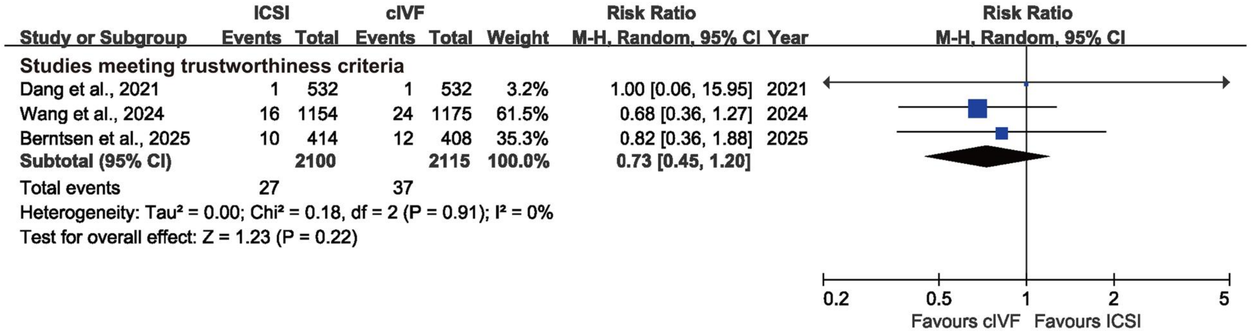

Supplementary Figure S14. **Forest plot of gestational hypertension rate in couples without severe male factor infertility.** cIVF, conventional IVF.
