# Supplementary material for: Does intracytoplasmic sperm injection outperform conventional in vitro fertilization in couples without severe male factor infertility? A systematic review and meta-analysis of randomized controlled trials
Source: Hum Reprod. 2026 May 22;41(7):1173–82. doi: 10.1093/humrep/deag066 (PMC13334920; doi:10.1093/humrep/deag066)
Supplement: deag066_Supplementary_Figure_S15 [file deag066_supplementary_figure_s15.pdf]

Human Reproduction, 2026, 1–2
https://doi.org/10.1093/humrep/deag066
Supplementary Data

human reproduction

OXFORD

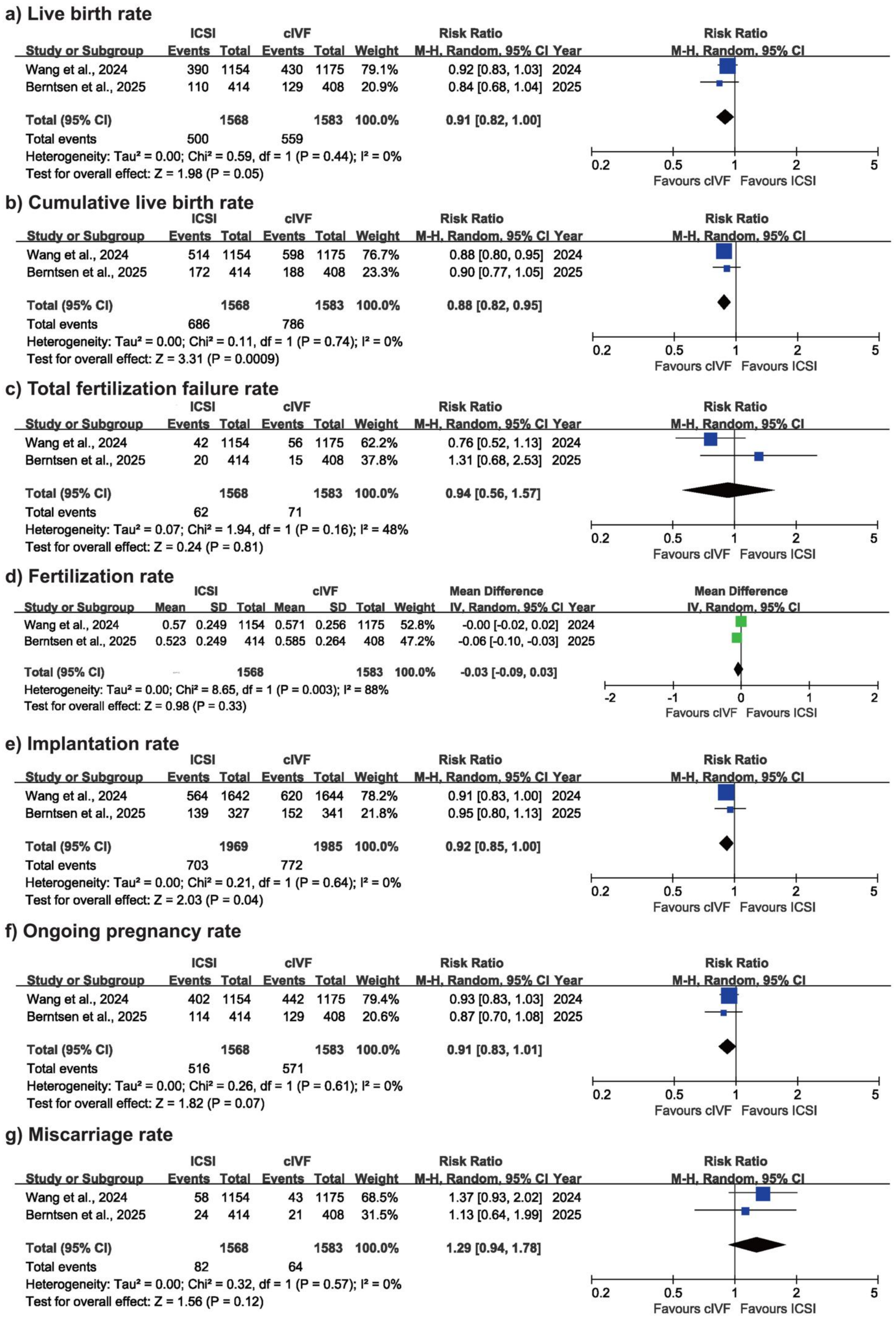

Supplementary Figure S15. Forest plot of livebirth rate, cumulative livebirth rate, total fertilization failure, fertilization rate, implantation rate, ongoing pregnancy rate, miscarriage rate, preterm birth rate, low birth weight rate, birth defect rate, multiple pregnancy rate, ectopic pregnancy rate, gestational diabetes rate, and gestational hypertension rate in couples with normal or non-severe male factor infertility. (**a**) Livebirth rate; (**b**) cumulative livebirth rate; (**c**) total fertilization failure; (**d**) fertilization rate; (**e**) implantation rate; (**f**) ongoing pregnancy rate; (**g**) miscarriage rate; (**h**) preterm birth rate; (**i**) low birth weight rate; (**j**) birth defect rate; (**k**) multiple pregnancy rate; (**l**) ectopic pregnancy rate; (**m**) gestational diabetes rate; (**n**) gestational hypertension rate.cIVF, conventional IVF.

Human Reproduction, 2026, 1–1
https://doi.org/10.1093/humrep/deag066
Supplementary Data

human reproduction

OXFORD

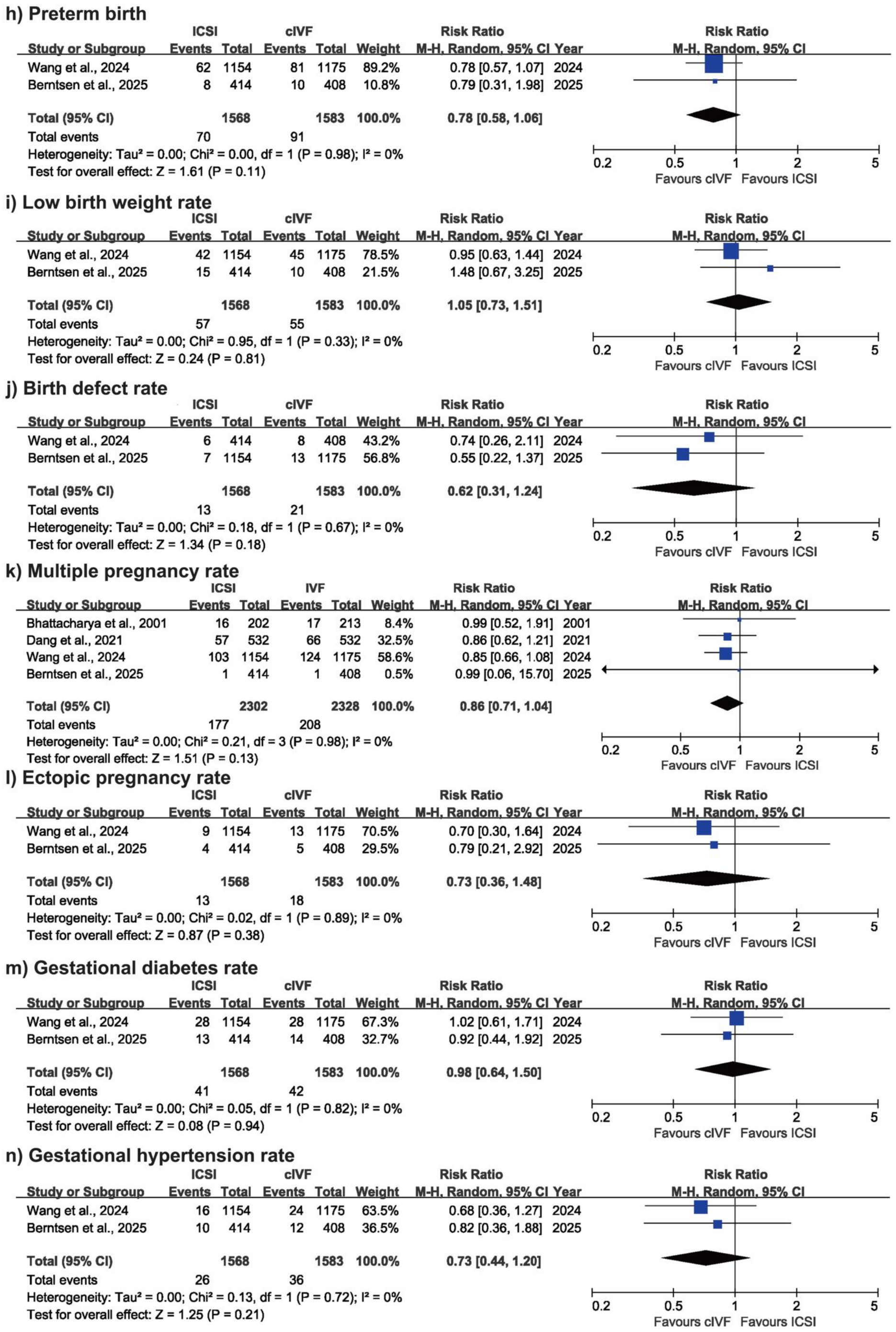

Supplementary Figure S15. (Continued)
