# Supplementary material for: Does intracytoplasmic sperm injection outperform conventional in vitro fertilization in couples without severe male factor infertility? A systematic review and meta-analysis of randomized controlled trials
Source: Hum Reprod. 2026 May 22;41(7):1173–82. doi: 10.1093/humrep/deag066 (PMC13334920; doi:10.1093/humrep/deag066)
Supplement: deag066_Supplementary_Figure_S17 [file deag066_supplementary_figure_s17.pdf]

Human Reproduction, 2026, 1–1
https://doi.org/10.1093/humrep/deag066
Supplementary Data

human reproduction
OXFORD

	ICSI		cIVF			Risk Ratio	
Study or Subgroup	Events	Total	Events	Total	Weight	M-H, Fixed, 95% CI	Year
Foong et al., 2006	15	30	14	30	17.1%	1.07 [0.63, 1.81]	2006
Dang et al., 2021	73	199	65	183	82.9%	1.03 [0.79, 1.35]	2021
**Total (95% CI)**		**229**		**213**	**100.0%**	**1.04 [0.82, 1.32]**	
Total events	88		79				

Heterogeneity: $Chi^2 = 0.02$, df = 1 (P = 0.90); $I^2 = 0\%$
Test for overall effect: Z = 0.32 (P = 0.75)

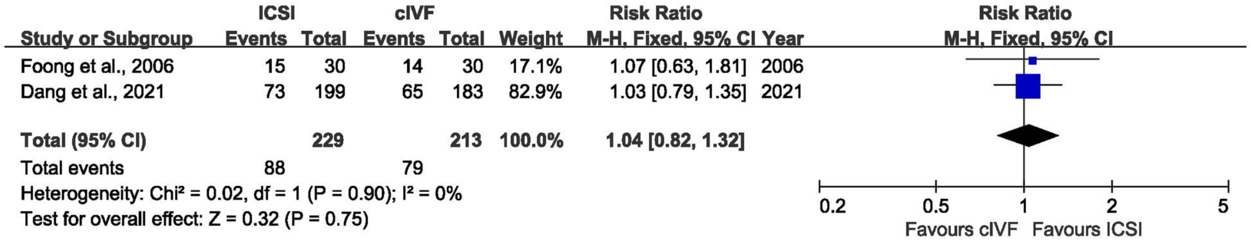

Supplementary Figure S17. Forest plot of live birth rate in couples with unexplained infertility. cIVF, conventional IVF.
