# Supplementary material for: Does intracytoplasmic sperm injection outperform conventional in vitro fertilization in couples without severe male factor infertility? A systematic review and meta-analysis of randomized controlled trials
Source: Hum Reprod. 2026 May 22;41(7):1173–82. doi: 10.1093/humrep/deag066 (PMC13334920; doi:10.1093/humrep/deag066)
Supplement: deag066_Supplementary_Figure_S20 [file deag066_supplementary_figure_s20.pdf]

Human Reproduction, 2026, 1–1
https://doi.org/10.1093/humrep/deag066
Supplementary Data

human reproduction

OXFORD

Study	ICSI Events	ICSI Total	cIVF Events	cIVF Total	RR	95%-CI	Weight
Foong et al. (2006)	15	30	14	30	1.07	[0.63; 1.81]	5.6%
Dang et al. (2021)	184	532	166	532	1.11	[0.93; 1.32]	29.4%
Wang et al. (2024)	390	1154	430	1175	0.92	[0.83; 1.03]	42.0%
Berntsen et al. (2025)	110	414	129	408	0.84	[0.68; 1.04]	22.9%
**Random effects model**		**2130**		**2145**	**0.96**	**[0.84; 1.10]**	**100.0%**

Heterogeneity: $I^2 = 37.4\%$, $\tau^2 = 0.0074$, $p = 0.1879$

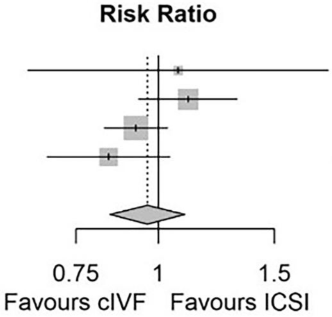

Supplementary Figure S20. Sensitivity analyses of live birth rate in couples without severe male factor infertility (using REML based estimator). cIVF, conventional IVF; REML, restricted maximum likelihood.
