# Supplementary material for: Does intracytoplasmic sperm injection outperform conventional in vitro fertilization in couples without severe male factor infertility? A systematic review and meta-analysis of randomized controlled trials
Source: Hum Reprod. 2026 May 22;41(7):1173–82. doi: 10.1093/humrep/deag066 (PMC13334920; doi:10.1093/humrep/deag066)
Supplement: deag066_Supplementary_Figure_S21 [file deag066_supplementary_figure_s21.pdf]

Human Reproduction, 2026, 1–1
https://doi.org/10.1093/humrep/deag066
Supplementary Data

human reproduction
OXFORD

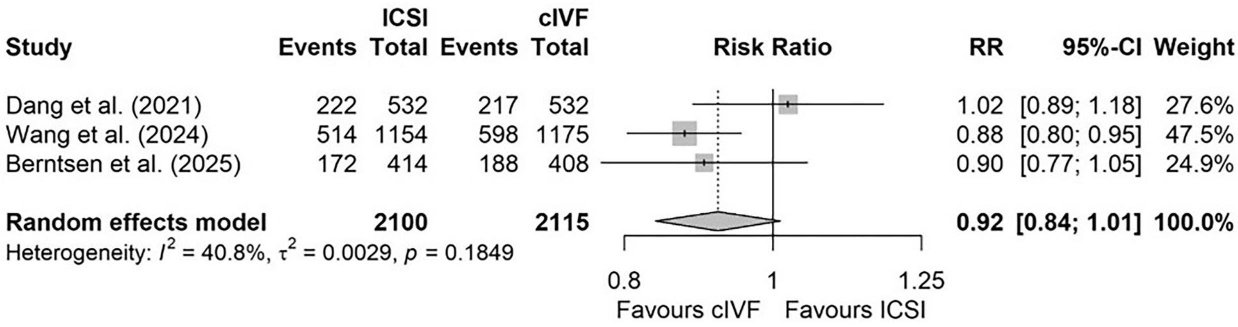

Supplementary Figure S21. **Sensitivity analyses of cumulative live birth rate in couples without severe male factor infertility (using REML-based estimator).** cIVF, conventional IVF; REML, restricted maximum likelihood.
