# Supplementary material for: Does intracytoplasmic sperm injection outperform conventional in vitro fertilization in couples without severe male factor infertility? A systematic review and meta-analysis of randomized controlled trials
Source: Hum Reprod. 2026 May 22;41(7):1173–82. doi: 10.1093/humrep/deag066 (PMC13334920; doi:10.1093/humrep/deag066)
Supplement: deag066_Supplementary_Table_S1 [file deag066_supplementary_table_s1.pdf]

Human Reproduction, 2026, 1–1
https://doi.org/10.1093/humrep/deag066
Supplementary Data

human reproduction

OXFORD

Supplementary Table S1. Search strategy.
PubMed (Controlled Vocabulary)
On 10 June 2025
Published by 31 May 2025

Step	Searches	Results
1	'Fertilization in Vitro'[Mesh]	43 554
2	'Sperm Injections, Intracytoplasmic'[Mesh]	8509
3	'Live Birth'[Mesh] OR 'Pregnancy Rate'[Mesh] OR 'Abortion, Spontaneous'[Mesh] OR 'Premature Birth'[Mesh] OR 'Stillbirth'[Mesh] OR 'Infant, Low Birth Weight'[Mesh] OR 'Infant, Very Low Birth Weight'[Mesh] OR 'Infant, Extremely Low Birth Weight'[Mesh] OR 'Congenital Abnormalities'[Mesh] OR 'Perinatal Death'[Mesh]	807 655
4	'Randomized Controlled Trial'[Publication Type] OR randomized [Title/Abstract] OR placebo[Title/Abstract] OR randomised[Title/Abstract] OR random[Title/Abstract] OR RCT[Title/Abstract]	1 557 929
5	Step 1 AND Step 2 AND Step 3 AND Step 4	**655**

PubMed (free-text Terms)
On 10 June 2025
Published by 31 May 2025

Step	Searches	Results
1	'In Vitro Fertilization' OR 'In Vitro Fertilizations' OR IVF OR 'Fertilization in Vitro' OR 'Fertilizations in Vitro' OR 'Test-Tube Fertilization' OR 'Test Tube Fertilization' OR 'Test-Tube Baby' OR 'Test-Tube Babies' OR 'Test Tube Baby' OR 'Test Tube Babies'	61 988
2	'intracytoplasmic sperm injection' OR 'intracytoplasmic sperm injections' OR ICSI	16 454
3	'Live-Birth Pregnancy Rate' OR 'Live-Birth Pregnancy Rates' OR 'Live Birth Pregnancy Rate' OR 'Live Birth Pregnancy Rates' OR 'Liveborn Child' OR 'Live Birth' OR 'Live Births' OR 'Live Birth Rate' OR 'Fertilization failure' OR 'Complete fertilization failure' OR 'Fertilization rate' OR 'Implantation rate' OR 'Pregnancy Rate' OR 'Pregnancy Rates' OR 'Clinical pregnancy' OR 'Clinical pregnancies' OR 'Ongoing clinical pregnancy' OR 'Ongoing pregnancy rate' OR 'Spontaneous Abortion' OR 'Spontaneous Abortions' OR 'Early Pregnancy Loss' OR 'Early Pregnancy Losses' OR Miscarriage OR Miscarriages OR 'Tubal Abortion' OR 'Tubal Abortions' OR 'Loss of pregnancy' OR 'pregnancy loss' OR 'spontaneous pregnancy loss' OR 'Premature Birth' OR 'Premature Births' OR 'Preterm Birth' OR 'Preterm Births' OR 'Pre-mature Birth' OR 'Pre-mature infant' OR 'Pre-maturity' OR 'Pre-term baby' OR 'Pre-term babies' OR 'Pre-term birth' OR 'Pre-term child' OR 'Pre-term infant' OR 'Pre-term infants' OR 'Pre-term neonate' OR 'Pre-term neonates' OR 'Pre-term newborn' OR 'Pre-term newborns' OR Premature OR 'Premature baby' OR 'Premature babies' OR 'Premature birth' OR 'Premature child' OR 'Premature childbirth' OR 'Premature infant' OR 'Premature infants' OR 'Premature neonate' OR 'Premature neonates' OR 'Premature newborn' OR 'Premature newborns' OR 'Premature syndrome' OR 'Preterm baby' OR 'Preterm babies' OR 'Preterm child' OR 'Preterm infant' OR 'Preterm infants' OR 'Preterm neonate' OR 'Preterm neonates' OR 'Preterm newborn' OR 'Preterm newborns' OR Prematurity OR Stillbirth OR 'Stillbirths' OR 'Still Birth' OR 'Still Births' OR 'Still Born Baby' OR 'Still Born Babies' OR 'Low Birth Weight' OR 'Low Birth Weights' OR 'Low-Birth-Weight' OR 'Very Low Birth Weight' OR 'Very-Low-Birth-Weight' OR 'Very Low Birth Weight' OR 'Extremely Low Birth Weight' OR 'Congenital Abnormality' OR Deformity OR Deformities OR 'Congenital Defect' OR 'Congenital Defects' OR 'Birth Defect' OR 'Birth Defects' OR 'Fetal Malformation' OR 'Fetal Malformations' OR 'Fetal Anomaly' OR 'Fetal Anomalies' OR 'Congenital Anomaly' OR 'Congenital Deformity' OR 'Development Anomaly' OR Malformation OR 'Malformative Disease' OR 'Congenital Malformation' OR 'Perinatal Death' OR 'Perinatal Deaths' OR 'Neonatal Death' OR 'Neonatal Deaths' OR 'New Born Death' OR 'New Born Deaths' OR 'Cumulative clinical pregnancy' OR 'Cumulative clinical pregnancies' OR 'Cumulative clinical pregnancy rate' OR 'Cumulative Live Birth Rate'	1 766 156
4	'Randomized Controlled Trial'[Publication Type] OR randomized[Title/Abstract] OR placebo[Title/Abstract] OR randomised[Title/Abstract] OR random[Title/Abstract] OR RCT[Title/Abstract]	1 557 929
5	Step 1 AND Step 2 AND Step 3 AND Step 4	**1330**

Human Reproduction, 2026, 1–1
https://doi.org/10.1093/humrep/deag066
Supplementary Data

human reproduction

OXFORD

Embase
On 10 June 2025
Published by 31 May 2025

Step	Searches	Results
1	‘in vitro fertilization’/exp	129 466
2	‘intracytoplasmic sperm injection’/exp	31 344
3	‘live birth’/exp OR ‘spontaneous abortion’/exp OR ‘prematurity’/exp OR ‘stillbirth’/exp OR ‘low birth weight’/exp OR ‘congenital malformation’/exp OR ‘newborn death’/exp	1 459 353
4	‘randomized controlled trail’:ab,ti OR ‘randomized’:ab,ti OR ‘placebo’:ab,ti OR ‘randomised’:ab,ti OR ‘random’:ab,ti OR rct:ab,ti	2 108 476
5	Step 1 AND Step 2 AND Step 3 AND Step 4	**1595**
6	‘extracorporeal fertilization’:ti,ab,kw OR ‘fertilization in vitro’:ti,ab,kw OR ‘fertilizations in vitro’:ti,ab,kw OR ‘in vitro fertilisation’:ti,ab,kw OR ‘in vitro fertilization’:ti,ab,kw OR ‘in vitro fertilizations’:ti,ab,kw OR ‘ivf’:ti,ab,kw OR ‘test tube baby’:ti,ab,kw OR ‘test tube babies’:ti,ab,kw OR ‘test-tube baby’:ti,ab,kw OR ‘test-tube babies’:ti,ab,kw OR ‘test tube fertilization’:ti,ab,kw OR ‘test tube fertilizations’:ti,ab,kw OR ‘test-tube fertilization’:ti,ab,kw OR ‘test-tube fertilizations’:ti,ab,kw	79 903
7	‘icsi’:ti,ab,kw OR ‘intracytoplasmic sperm injections’:ti,ab,kw OR ‘intracytoplasmic sperm injection’:ti,ab,kw	28 382
8	‘live-birth pregnancy rate’:ti,ab,kw OR ‘live-birth pregnancy rates’:ti,ab,kw OR ‘live birth pregnancy rate’:ti,ab,kw OR ‘live birth pregnancy rates’:ti,ab,kw OR ‘liveborn child’:ti,ab,kw OR ‘live birth’:ti,ab,kw OR ‘live births’:ti,ab,kw OR ‘live birth rate’:ti,ab,kw OR ‘fertilization failure’:ti,ab,kw OR ‘complete fertilization failure’:ti,ab,kw OR ‘fertilization rate’:ti,ab,kw OR ‘Implantation rate’:ti,ab,kw OR ‘pregnancy rate’:ti,ab,kw OR ‘pregnancy rates’:ti,ab,kw OR ‘clinical pregnancy’:ti,ab,kw OR ‘clinical pregnancies’:ti,ab,kw OR ‘ongoing clinical pregnancy’:ti,ab,kw OR ‘ongoing pregnancy rate’:ti,ab,kw OR ‘spontaneous abortion’:ti,ab,kw OR ‘spontaneous abortions’:ti,ab,kw OR ‘early pregnancy loss’:ti,ab,kw OR ‘early pregnancy losses’:ti,ab,kw OR miscarriage: ti,ab,kw OR miscarriages: ti,ab,kw OR ‘tubal abortion’:ti,ab,kw OR ‘tubal abortions’:ti,ab,kw OR ‘loss of pregnancy’:ti,ab,kw OR ‘pregnancy loss’:ti,ab,kw OR ‘spontaneous pregnancy loss’:ti,ab,kw OR ‘premature births’:ti,ab,kw OR ‘preterm birth’:ti,ab,kw OR ‘preterm births’:ti,ab,kw OR ‘pre-mature birth’:ti,ab,kw OR ‘pre-mature infant’:ti,ab,kw OR ‘pre-maturity’:ti,ab,kw OR ‘pre-term baby’:ti,ab,kw OR ‘pre-term babies’:ti,ab,kw OR ‘pre-term birth’:ti,ab,kw OR ‘pre-term child’:ti,ab,kw OR ‘pre-term infant’:ti,ab,kw OR ‘pre-term infants’:ti,ab,kw OR ‘pre-term neonate’:ti,ab,kw OR ‘pre-term neonates’:ti,ab,kw OR ‘pre-term newborn’:ti,ab,kw OR ‘pre-term newborns’:ti,ab,kw OR premature: ti,ab,kw OR ‘premature baby’:ti,ab,kw OR ‘premature babies’:ti,ab,kw OR ‘premature birth’:ti,ab,kw OR ‘premature child’:ti,ab,kw OR ‘premature childbirth’:ti,ab,kw OR ‘premature infant’:ti,ab,kw OR ‘premature infants’:ti,ab,kw OR ‘premature neonate’:ti,ab,kw OR ‘premature neonates’:ti,ab,kw OR ‘premature newborn’:ti,ab,kw OR ‘premature newborns’:ti,ab,kw OR ‘premature syndrome’:ti,ab,kw OR ‘preterm baby’:ti,ab,kw OR ‘preterm babies’:ti,ab,kw OR ‘preterm child’:ti,ab,kw OR ‘preterm infant’:ti,ab,kw OR ‘preterm infants’:ti,ab,kw OR ‘preterm neonate’:ti,ab,kw OR ‘preterm neonates’:ti,ab,kw OR ‘preterm newborn’:ti,ab,kw OR ‘preterm newborns’:ti,ab,kw OR prematurity:ti,ab,kw OR stillbirth:ti,ab,kw OR ‘stillbirths’:ti,ab,kw OR ‘still birth’:ti,ab,kw OR ‘still births’:ti,ab,kw OR ‘still born baby’:ti,ab,kw OR ‘still born babies’:ti,ab,kw OR ‘low birth weight’:ti,ab,kw OR ‘low birth weights’:ti,ab,kw OR ‘low-birth-weight’:ti,ab,kw OR ‘very-low-birth-weight’:ti,ab,kw OR ‘very low birth weight’:ti,ab,kw OR ‘extremely low birth weight’:ti,ab,kw OR ‘congenital abnormality’:ti,ab,kw OR deformity:ti,ab,kw OR deformities:ti,ab,kw OR ‘congenital defect’:ti,ab,kw OR ‘congenital defects’:ti,ab,kw OR ‘birth defect’:ti,ab,kw OR ‘birth defects’:ti,ab,kw OR ‘fetal malformation’:ti,ab,kw OR ‘fetal malformations’:ti,ab,kw OR ‘fetal anomaly’:ti,ab,kw OR ‘fetal anomalies’:ti,ab,kw OR ‘congenital anomaly’:ti,ab,kw OR ‘congenital deformity’:ti,ab,kw OR ‘development anomaly’:ti,ab,kw OR malformation:ti,ab,kw OR ‘malformative disease’:ti,ab,kw OR ‘congenital malformation’:ti,ab,kw OR ‘perinatal death’:ti,ab,kw OR ‘perinatal deaths’:ti,ab,kw OR ‘neonatal death’:ti,ab,kw OR ‘neonatal deaths’:ti,ab,kw OR ‘new born death’:ti,ab,kw OR ‘new born deaths’:ti,ab,kw OR ‘cumulative clinical pregnancy’:ti,ab,kw OR ‘cumulative clinical pregnancies’:ti,ab,kw OR ‘cumulative clinical pregnancy rate’:ti,ab,kw OR ‘cumulative live birth rate’:ti,ab,kw	740 337
9	Step 6 AND Step 7 AND Step 8 AND Step 4	**2449**

Human Reproduction, 2026, 1–1
https://doi.org/10.1093/humrep/deag066
Supplementary Data

human reproduction
OXFORD

The Cochrane Library(Trails)
On 10 June 2025
Published by 31 May 2025

Step	Searches	Results
1	MeSH descriptor: [Fertilization in Vitro] explode all trees	2865
2	MeSH descriptor: [Sperm Injections, Intracytoplasmic] explode all trees	754
3	MeSH descriptor: [Live Birth] explode all trees	431
4	MeSH descriptor: [Pregnancy Rate] explode all trees	2296
5	MeSH descriptor: [Abortion, Spontaneous] explode all trees	1254
6	MeSH descriptor: [Premature Birth] explode all trees	2460
7	MeSH descriptor: [Stillbirth] explode all trees	205
8	MeSH descriptor: [Infant, Low Birth Weight] explode all trees	2809
9	MeSH descriptor: [Infant, Very Low Birth Weight] explode all trees	1300
10	MeSH descriptor: [Infant, Extremely Low Birth Weight] explode all trees	172
11	MeSH descriptor: [Congenital Abnormalities] explode all trees	9586
12	MeSH descriptor: [Perinatal Death] explode all trees	142
13	Step 3 OR Step 4 OR Step 5 OR Step 6 OR Step 7 OR Step 8 OR Step 9 OR Step 10 OR Step 11 OR Step 12	18092
14	Step 1 AND Step 2 AND Step 13	**465**
15	(In Vitro Fertilization):ti,ab,kw OR (In Vitro Fertilizations):ti,ab,kw OR (IVF):ti,ab,kw OR (Fertilization in Vitro):ti,ab,kw OR (Fertilizations in Vitro):ti,ab,kw OR (Test-Tube Fertilization):ti,ab,kw OR (Test-Tube Fertilizations):ti,ab,kw OR (Test Tube Fertilization):ti,ab,kw OR (Test Tube Fertilizations):ti,ab,kw OR (Test-Tube Baby):ti,ab,kw OR (Test-Tube Babies):ti,ab,kw OR (Test Tube Baby):ti,ab,kw OR (Test Tube Babies):ti,ab,kw OR (Extracpeal Fertilization):ti,ab,kw	9428
16	(intracytoplasmic sperm injection):ti,ab,kw OR (intracytoplasmic sperm injections):ti,ab,kw OR (ICSI):ti,ab,kw	4426
17	(Live-Birth Pregnancy Rate):ti,ab,kw OR (Live-Birth Pregnancy Rates):ti,ab,kw OR (Live Birth Pregnancy Rate):ti,ab,kw OR (Live Birth Pregnancy Rates):ti,ab,kw OR (Liveborn Child):ti,ab,kw OR (Liveborn Progeny):ti,ab,kw OR (Live Birth):ti,ab,kw OR (Live Births):ti,ab,kw OR (Live Birth Rate):ti,ab,kw (Fertilization failure):ti,ab,kw OR (Complete fertilization failure):ti,ab,kw OR (Fertilization rate):ti,ab,kw OR (Implantation rate):ti,ab,kw OR (Pregnancy Rate):ti,ab,kw OR (Pregnancy Rates):ti,ab,kw OR (Clinical pregnancy):ti,ab,kw OR (Clinical pregnancies):ti,ab,kw OR (Ongoing clinical pregnancy):ti,ab,kw OR (Ongoing clinical pregnancies):ti,ab,kw OR (Ongoing pregnancy rate):ti,ab,kw OR (Spontaneous Abortion):ti,ab,kw OR (Spontaneous Abortions):ti,ab,kw OR (Early Pregnancy Loss):ti,ab,kw OR (Early Pregnancy Losses):ti,ab,kw OR (Miscarriage):ti,ab,kw OR (Miscarriages):ti,ab,kw OR (Tubal Abortion):ti,ab,kw OR (Tubal Abortions):ti,ab,kw OR (Loss of a pregnancy):ti,ab,kw OR (Loss of pregnancy):ti,ab,kw OR (loss of the pregnancy):ti,ab,kw OR (pregnancy loss):ti,ab,kw OR (spontaneous pregnancy loss):ti,ab,kw OR (Premature Birth):ti,ab,kw OR (Premature Births):ti,ab,kw OR (Preterm Birth):ti,ab,kw OR (Preterm Births):ti,ab,kw OR (Pre-mature Birth):ti,ab,kw OR (Pre-mature infant):ti,ab,kw OR (Pre-maturity):ti,ab,kw OR (Pre-term baby):ti,ab,kw OR (Pre-term babies):ti,ab,kw OR (Pre-term birth):ti,ab,kw OR (Pre-term child):ti,ab,kw OR (Pre-term infant):ti,ab,kw OR (Pre-term infants):ti,ab,kw OR (Pre-term neonate):ti,ab,kw OR (Pre-term neonates):ti,ab,kw OR (Pre-term newborn):ti,ab,kw OR (Pre-term newborns):ti,ab,kw OR (Premature):ti,ab,kw OR (Premature baby):ti,ab,kw OR (Premature babies):ti,ab,kw OR (Premature birth):ti,ab,kw OR (Premature child):ti,ab,kw OR (Premature childbirth):ti,ab,kw OR (Premature infant):ti,ab,kw OR (Premature infants):ti,ab,kw OR (Premature neonate):ti,ab,kw OR (Premature neonates):ti,ab,kw OR (Premature newborn):ti,ab,kw OR (Premature newborns):ti,ab,kw OR (Premature syndrome):ti,ab,kw OR (Preterm baby):ti,ab,kw OR (Preterm babies):ti,ab,kw OR (Preterm child):ti,ab,kw OR (Preterm infant):ti,ab,kw OR (Preterm infants):ti,ab,kw OR (Preterm neonate):ti,ab,kw OR (Preterm neonates):ti,ab,kw OR (Preterm newborn):ti,ab,kw OR (Preterm newborns):ti,ab,kw OR (Prematurity):ti,ab,kw OR (Stillbirth):ti,ab,kw OR (Stillbirths):ti,ab,kw OR (Still Birth):ti,ab,kw OR (Still Births):ti,ab,kw OR (Still Born Baby):ti,ab,kw OR (Still Born Babies):ti,ab,kw OR (Low Birth Weight):ti,ab,kw OR (Low Birth Weights):ti,ab,kw OR (Low-Birth-Weight):ti,ab,kw OR (Very Low Birth Weight):ti,ab,kw OR (Very-Low-Birth-Weight):ti,ab,kw OR (Very Low Birth Weight):ti,ab,kw OR (Extremely Low Birth Weight):ti,ab,kw OR (Congenital Abnormality):ti,ab,kw OR (Deformity):ti,ab,kw OR (Deformities):ti,ab,kw OR (Congenital Defect):ti,ab,kw OR (Congenital Defects):ti,ab,kw OR (Birth Defect):ti,ab,kw OR (Birth Defects):ti,ab,kw OR (Fetal Malformation):ti,ab,kw OR (Fetal Malformations):ti,ab,kw OR (Fetal Anomaly):ti,ab,kw OR (Fetal Anomalies):ti,ab,kw OR (Congenital Anomaly):ti,ab,kw OR (Congenital Deformity):ti,ab,kw OR (Development Anomaly):ti,ab,kw OR (Malformation):ti,ab,kw OR (Malformative Disease):ti,ab,kw OR (Congenital Malformation):ti,ab,kw OR (Perinatal Death):ti,ab,kw OR (Perinatal Deaths):ti,ab,kw OR (Neonatal Death):ti,ab,kw OR (Neonatal Deaths):ti,ab,kw OR (New Born Death):ti,ab,kw OR (New Born Deaths):ti,ab,kw OR (Cumulative clinical pregnancy):ti,ab,kw OR (Cumulative clinical pregnancies):ti,ab,kw OR (Cumulative clinical pregnancy rate):ti,ab,kw OR (Cumulative Live Birth Rate):ti,ab,kw	95 708
18	Step 15 AND Step 16 AND Step 17	**2646**

Human Reproduction, 2026, 1–1
https://doi.org/10.1093/humrep/deag066
Supplementary Data

human reproduction OXFORD

Web of Science(All Databases)
On 10 June 2025
Published by 31 May 2025

Step	Searches	Results
1	TS=('In Vitro Fertilization' OR 'In Vitro Fertilizations' OR IVF OR 'Fertilization in Vitro' OR 'Fertilizations in Vitro' OR 'Test-Tube Fertilization' OR 'Test-Tube Fertilizations' OR 'Test Tube Fertilization' OR 'Test Tube Fertilizations' OR 'Test-Tube Baby' OR 'Test-Tube Babies' OR 'Test Tube Baby' OR 'Test Tube Babies' OR 'Extracpeal Fertilization')	97 578
2	TS=('Sperm Injections, Intracytoplasmic' OR 'intracytoplasmic sperm injection' OR 'intracytoplasmic sperm injections' OR ICSI)	29 632
3	TS=('Live-Birth Pregnancy Rate' OR 'Live-Birth Pregnancy Rates' OR 'Live Birth Pregnancy Rate' OR 'Live Birth Pregnancy Rates' OR 'Liveborn Child' OR 'Liveborn Progeny' OR 'Live Birth' OR 'Live Births' OR 'Live Birth Rate' OR 'Fertilization failure' OR 'Complete fertilization failure' OR 'Fertilization rate' OR 'Implantation rate' OR 'Pregnancy Rate' OR 'Pregnancy Rates' OR 'Clinical pregnancy' OR 'Clinical pregnancies' OR 'Ongoing clinical pregnancy' OR 'Ongoing clinical pregnancies' OR 'Ongoing pregnancy rate' OR 'Spontaneous Abortion' OR 'Spontaneous Abortions' OR 'Early Pregnancy Loss' OR 'Early Pregnancy Losses' OR Miscarriage OR 'Miscarriages' OR 'Tubal Abortion' OR 'Tubal Abortions' OR 'Loss of a pregnancy' OR 'Loss of pregnancy' OR 'loss of the pregnancy' OR 'pregnancy loss' OR 'spontaneous pregnancy loss' OR 'Premature Birth' OR 'Premature Births' OR 'Preterm Birth' OR 'Preterm Births' OR 'Pre-mature Birth' OR 'Pre-mature infant' OR 'Pre-maturity' OR 'Pre-term baby' OR 'Pre-term babies' OR 'Pre-term birth' OR 'Pre-term child' OR 'Pre-term infant' OR 'Pre-term infants' OR 'Pre-term neonate' OR 'Pre-term neonates' OR 'Pre-term newborn' OR 'Pre-term newborns' OR Premature OR 'Premature baby' OR 'Premature babies' OR 'Premature birth' OR 'Premature child' OR 'Premature child-birth' OR 'Premature infant' OR 'Premature infants' OR 'Premature neonate' OR 'Premature neonates' OR 'Premature newborn' OR 'Premature newborns' OR 'Premature syndrome' OR 'Preterm baby' OR 'Preterm babies' OR 'Preterm child' OR 'Preterm infant' OR 'Preterm infants' OR 'Preterm neonate' OR 'Preterm neonates' OR 'Preterm newborn' OR 'Preterm newborns' OR Prematurity OR Stillbirth OR 'Stillbirths' OR 'Still Birth' OR 'Still Births' OR 'Still Born Baby' OR 'Still Born Babies' OR 'Low Birth Weight' OR 'Low Birth Weights' OR 'Low-Birth-Weight' OR 'Very Low Birth Weight' OR 'Very-Low-Birth-Weight' OR 'Very Low Birth Weight' OR 'Extremely Low Birth Weight' OR 'Congenital Abnormality' OR Deformity OR Deformities OR 'Congenital Defect' OR 'Congenital Defects' OR 'Birth Defect' OR 'Birth Defects' OR 'Fetal Malformation' OR 'Fetal Malformations' OR 'Fetal Anomaly' OR 'Fetal Anomalies' OR 'Congenital Anomaly' OR 'Congenital Deformity' OR 'Development Anomaly' OR Malformation OR 'Malformative Disease' OR 'Congenital Malformation' OR 'Perinatal Death' OR 'Perinatal Deaths' OR 'Neonatal Death' OR 'Neonatal Deaths' OR 'New Born Death' OR 'New Born Deaths' OR 'Cumulative clinical pregnancy' OR 'Cumulative clinical pregnancies' OR 'Cumulative clinical pregnancy rate' OR 'Cumulative Live Birth Rate')	1 047 966
4	TS=('randomized controlled trail' OR randomized OR placebo OR randomised OR random OR RCT)	3 478 913
5	Step 1 AND Step 2 AND Step 3 AND Step 4	**1964**

Human Reproduction, 2026, 1–1
https://doi.org/10.1093/humrep/deag066
Supplementary Data

human reproduction
OXFORD

ProQuest (23 Databases)
On 10 June 2025
Published by 31 May 2025

Step	Searches	Results
1	AB, TI('In Vitro Fertilization' OR 'In Vitro Fertilizations' OR IVF OR 'Fertilization in Vitro' OR 'Fertilizations in Vitro' OR 'Test-Tube Fertilization' OR 'Test-Tube Fertilizations' OR 'Test Tube Fertilization' OR 'Test Tube Fertilizations' OR 'Test-Tube Baby' OR 'Test-Tube Babies' OR 'Test Tube Baby' OR 'Test Tube Babies' OR 'Extracpeal Fertilization')	26 103
2	AB, TI('Sperm Injections, Intracytoplasmic' OR 'intracytoplasmic sperm injection' OR 'intracytoplasmic sperm injections' OR ICSI)	7128
3	AB, TI('Live-Birth Pregnancy Rate' OR 'Live-Birth Pregnancy Rates' OR 'Live Birth Pregnancy Rate' OR 'Live Birth Pregnancy Rates' OR 'Liveborn Child' OR 'Liveborn Progeny' OR 'Live Birth' OR 'Live Births' OR 'Live Birth Rate' OR 'Fertilization failure' OR 'Complete fertilization failure' OR 'Fertilization rate' OR 'Implantation rate' OR 'Pregnancy Rate' OR 'Pregnancy Rates' OR 'Clinical pregnancy' OR 'Clinical pregnancies' OR 'Ongoing clinical pregnancy' OR 'Ongoing clinical pregnancies' OR 'Ongoing pregnancy rate' OR 'Spontaneous Abortion' OR 'Spontaneous Abortions' OR 'Early Pregnancy Loss' OR 'Early Pregnancy Losses' OR Miscarriage OR Miscarriages OR 'Tubal Abortion' OR 'Tubal Abortions' OR 'Loss of a pregnancy' OR 'Loss of pregnancy' OR 'loss of the pregnancy' OR 'pregnancy loss' OR 'spontaneous pregnancy loss' OR 'Premature Birth' OR 'Premature Births' OR 'Preterm Birth' OR 'Preterm Births' OR 'Pre-mature Birth' OR 'Pre-mature infant' OR 'Pre-maturity' OR 'Pre-term baby' OR 'Pre-term babies' OR 'Pre-term birth' OR 'Pre-term child' OR 'Pre-term infant' OR 'Pre-term infants' OR 'Pre-term neonate' OR 'Pre-term neonates' OR 'Pre-term newborn' OR 'Pre-term newborns' OR 'Premature' OR 'Premature baby' OR 'Premature babies' OR 'Premature birth' OR 'Premature child' OR 'Premature childbirth' OR 'Premature infant' OR 'Premature infants' OR 'Premature neonate' OR 'Premature neonates' OR 'Premature newborn' OR 'Premature newborns' OR 'Premature syndrome' OR 'Preterm baby' OR 'Preterm babies' OR 'Preterm child' OR 'Preterm infant' OR 'Preterm infants' OR 'Preterm neonate' OR 'Preterm neonates' OR 'Preterm newborn' OR 'Preterm newborns' OR Prematurity OR Stillbirth OR 'Stillbirths' OR 'Still Birth' OR 'Still Births' OR 'Still Born Baby' OR 'Still Born Babies' OR 'Low Birth Weight' OR 'Low Birth Weights' OR 'Low-Birth-Weight' OR 'Very Low Birth Weight' OR 'Very-Low-Birth-Weight' OR 'Very Low Birth Weight' OR 'Extremely Low Birth Weight' OR 'Congenital Abnormality' OR 'Deformity' OR 'Deformities' OR 'Congenital Defect' OR 'Congenital Defects' OR 'Birth Defect' OR 'Birth Defects' OR 'Fetal Malformation' OR 'Fetal Malformations' OR 'Fetal Anomaly' OR 'Fetal Anomalies' OR 'Congenital Anomaly' OR 'Congenital Deformity' OR 'Development Anomaly' OR Malformation OR 'Malformative Disease' OR 'Congenital Malformation' OR 'Perinatal Death' OR 'Perinatal Deaths' OR 'Neonatal Death' OR 'Neonatal Deaths' OR 'New Born Death' OR 'New Born Deaths' OR 'Cumulative clinical pregnancy' OR 'Cumulative clinical pregnancies' OR 'Cumulative clinical pregnancy rate' 'Cumulative Live Birth Rate')	289 930
4	AB, TI('randomized controlled trail' OR randomized OR placebo OR randomised OR random OR RCT)	1 156 518
5	Step 1 AND Step 2 AND Step 3 AND Step 4	**356**

Human Reproduction, 2026, 1–1
https://doi.org/10.1093/humrep/deag066
Supplementary Data

human reproduction

OXFORD

MEDLINE [Ovid]
On 10 June 2025
Published by 31 May 2025

Step	Searches	Results
1	(In Vitro Fertilization or In Vitro Fertilizations or IVF or Fertilization in Vitro or Fertilizations in Vitro or Test-Tube Fertilization or Test-Tube Fertilizations or Test Tube Fertilization or Test Tube Fertilizations or Test-Tube Baby or Test-Tube Babies or Test Tube Baby or Test Tube Babies or Extracpeal Fertilization).ti, ab, kw.	45 321
2	(Sperm Injections, Intracytoplasmic or intracytoplasmic sperm injection or intracytoplasmic sperm injections or ICSI).ti, ab, kw.	14 551
3	(Live-Birth Pregnancy Rate or Live-Birth Pregnancy Rates or Live Birth Pregnancy Rate or Live Birth Pregnancy Rates or Liveborn Child or Liveborn Progeny or Live Birth or Live Births or Live Birth Rate or Fertilization failure or Complete fertilization failure or Fertilization rate or Implantation rate or Pregnancy Rate or Pregnancy Rates or Clinical pregnancy or Clinical pregnancies or Ongoing clinical pregnancy or Ongoing clinical pregnancies or Ongoing pregnancy rate or Spontaneous Abortion or Spontaneous Abortions or Early Pregnancy Loss or Early Pregnancy Losses or Miscarriage or Miscarriages or Tubal Abortion or Tubal Abortions or Loss of a pregnancy or Loss of pregnancy or loss of the pregnancy or pregnancy loss or spontaneous pregnancy loss or Premature Birth or Premature Births or Preterm Birth or Preterm Births or Pre-mature Birth or Pre-mature infant or Pre-maturity or Pre-term baby or Pre-term babies or Pre-term birth or Pre-term child or Pre-term infant or Pre-term infants or Pre-term neonate or Pre-term neonates or Pre-term newborn or Pre-term newborns or Premature or Premature baby or Premature babies or Premature birth or Premature child or Premature childbirth or Premature infant or Premature infants or Premature neonate or Premature neonates or Premature newborn or Premature newborns or Premature syndrome or Preterm baby or Preterm babies or Preterm child or Preterm infant or Preterm infants or Preterm neonate or Preterm neonates or Preterm newborn or Preterm newborns or Prematurity or Stillbirth or Stillbirths or Still Birth or Still Births or Still Born Baby or Still Born Babies or Low Birth Weight or Low Birth Weights or Low-Birth-Weight or Very Low Birth Weight or Very-Low-Birth-Weight or Very Low Birth Weight or Extremely Low Birth Weight or Congenital Abnormality or Deformity or Deformities or Congenital Defect or Congenital Defects or Birth Defect or Birth Defects or Fetal Malformation or Fetal Malformations or Fetal Anomaly or Fetal Anomalies or Congenital Anomaly or Congenital Deformity or Development Anomaly or Malformation or Malformative Disease or Congenital Malformation or Perinatal Death or Perinatal Deaths or Neonatal Death or Neonatal Deaths or New Born Death or New Born Deaths or Cumulative clinical pregnancy or Cumulative clinical pregnancies or Cumulative clinical pregnancy rate or Cumulative Live Birth Rate) .ti, ab, kw.	516 463
4	(randomized controlled trail or randomized or placebo or randomised or random or RCT).ti, ab, kw.	1 379 076
5	Step 1 AND Step 2 AND Step 3 AND Step 4	**1022**

Human Reproduction, 2026, 1–1
https://doi.org/10.1093/humrep/deag066
Supplementary Data

human reproduction

OXFORD

Scopus
On 10 June 2025
Published by 31 May 2025

Step	Searches	Results
1	TITLE-ABS-KEY('In Vitro Fertilization' OR 'In Vitro Fertilizations' OR IVF OR 'Fertilization in Vitro' OR 'Fertilizations in Vitro' OR 'Test-Tube Fertilization' OR 'Test-Tube Fertilizations' OR 'Test Tube Fertilization' OR 'Test Tube Fertilizations' OR 'Test-Tube Baby' OR 'Test-Tube Babies' OR 'Test Tube Baby' OR 'Test Tube Babies' OR 'Extracpeal Fertilization')	83 976
2	TITLE-ABS-KEY('Sperm Injections, Intracytoplasmic' OR 'intracytoplasmic sperm injection' OR 'intracytoplasmic sperm injections' OR ICSI)	26 359
3	TITLE-ABS-KEY('Live-Birth Pregnancy Rate' OR 'Live-Birth Pregnancy Rates' OR 'Live Birth Pregnancy Rate' OR 'Live Birth Pregnancy Rates' OR 'Liveborn Child' OR 'Liveborn Progeny' OR 'Live Birth' OR 'Live Births' OR 'Live Birth Rate' OR 'Fertilization failure' OR 'Complete fertilization failure' OR 'Fertilization rate' OR 'Implantation' OR 'Pregnancy Rate' OR 'Pregnancy Rates' OR 'Clinical pregnancy' OR 'Clinical pregnancies' OR 'Ongoing clinical pregnancy' OR 'Ongoing clinical pregnancies' OR 'Ongoing pregnancy rate' OR 'Spontaneous Abortion' OR 'Spontaneous Abortions' OR 'Early Pregnancy Loss' OR 'Early Pregnancy Losses' OR Miscarriage OR Miscarriages OR 'Tubal Abortion' OR 'Tubal Abortions' OR 'Loss of a pregnancy' OR 'Loss of pregnancy' OR 'loss of the pregnancy' OR 'pregnancy loss' OR 'spontaneous pregnancy loss' OR 'Premature Birth' OR 'Premature Births' OR 'Preterm Birth' OR 'Preterm Births' OR 'Pre-mature Birth' OR 'Pre-mature infant' OR 'Pre-maturity' OR 'Pre-term baby' OR 'Pre-term babies' OR 'Pre-term birth' OR 'Pre-term child' OR 'Pre-term infant' OR 'Pre-term infants' OR 'Pre-term neonate' OR 'Pre-term neonates' OR 'Pre-term newborn' OR 'Pre-term newborns' OR 'Premature' OR 'Premature baby' OR 'Premature babies' OR 'Premature birth' OR 'Premature child' OR 'Premature childbirth' OR 'Premature infant' OR 'Premature infants' OR 'Premature neonate' OR 'Premature neonates' OR 'Premature newborn' OR 'Premature newborns' OR 'Premature syndrome' OR 'Preterm baby' OR 'Preterm babies' OR 'Preterm child' OR 'Preterm infant' OR 'Preterm infants' OR 'Preterm neonate' OR 'Preterm neonates' OR 'Preterm newborn' OR 'Preterm newborns' OR Prematurity OR Stillbirth OR 'Stillbirths' OR 'Still Birth' OR 'Still Births' OR 'Still Born Baby' OR 'Still Born Babies' OR 'Low Birth Weight' OR 'Low Birth Weights' OR 'Low-Birth-Weight' OR 'Very Low Birth Weight' OR 'Very-Low-Birth-Weight' OR 'Very Low Birth Weight' OR 'Extremely Low Birth Weight' OR 'Congenital Abnormality' OR 'Deformity' OR 'Deformities' OR 'Congenital Defect' OR 'Congenital Defects' OR 'Birth Defect' OR 'Birth Defects' OR 'Fetal Malformation' OR 'Fetal Malformations' OR 'Fetal Anomaly' OR 'Fetal Anomalies' OR 'Congenital Anomaly' OR 'Congenital Deformity' OR 'Development Anomaly' OR Malformation OR 'Malformative Disease' OR 'Congenital Malformation' OR 'Perinatal Death' OR 'Perinatal Deaths' OR 'Neonatal Death' OR 'Neonatal Deaths' OR 'New Born Death' OR 'New Born Deaths' OR 'Cumulative clinical pregnancy' OR 'Cumulative clinical pregnancies' OR 'Cumulative clinical pregnancy rate' OR 'Cumulative Live Birth Rate')	1 605 116
4	TITLE-ABS-KEY('randomized controlled trail' OR randomized OR placebo OR randomised OR random OR RCT)	3 194 699
5	Step 1 AND Step 2 AND Step 3 AND Step 4	**2001**

Human Reproduction, 2026, 1–1
https://doi.org/10.1093/humrep/deag066
Supplementary Data

CINAHL Plus[EBSCO]
On 10 June 2025
Published by 31 May 2025

Step	Searches	Results
1	In Vitro Fertilization OR In Vitro Fertilizations OR IVF OR Fertilization in Vitro OR Fertilizations in Vitro OR Test-Tube Fertilization OR Test-Tube Fertilizations OR Test Tube Fertilization OR Test Tube Fertilizations OR Test-Tube Baby OR Test-Tube Babies OR Test Tube Baby OR Test Tube Babies OR Extracpeal Fertilization	11 833
2	intracytoplasmic sperm injection OR intracytoplasmic sperm injections OR ICSI	2612
3	Live-Birth Pregnancy Rate OR Live-Birth Pregnancy Rates OR Live Birth Pregnancy Rate OR Live Birth Pregnancy Rates Liveborn Child OR Liveborn Progeny OR Live Birth OR Live Births OR Live Birth Rate OR Fertilization failure OR Complete fertilization failure OR Fertilization rate OR Implantation Rate OR Pregnancy Rate OR Pregnancy Rates OR Clinical pregnancy OR Clinical pregnancies OR Ongoing clinical pregnancy OR Ongoing clinical pregnancies OR Ongoing pregnancy rate OR Spontaneous Abortion OR Spontaneous Abortions OR Early Pregnancy Loss OR Early Pregnancy Losses OR Miscarriage OR Miscarriages OR Tubal Abortion OR Tubal Abortions OR Loss of a pregnancy OR Loss of pregnancy OR loss of the pregnancy OR pregnancy loss OR spontaneous pregnancy loss OR Premature Birth OR Premature Births OR Preterm Birth OR Preterm Births OR Pre-mature Birth OR Pre-mature infant OR Pre-maturity OR Pre-term baby OR Pre-term babies OR Pre-term birth OR Pre-term child OR Pre-term infant OR Pre-term infants OR Pre-term neonate OR Pre-term neonates OR Pre-term newborn OR Pre-term newborns OR Premature OR Premature baby OR Premature babies OR Premature birth OR Premature child OR Premature childbirth OR Premature infant OR Premature infants OR Premature neonate OR Premature neonates OR Premature newborn OR Premature newborns OR Premature syndrome OR Preterm baby OR Preterm babies OR Preterm child OR Preterm infant OR Preterm infants OR Preterm neonate OR Preterm neonates OR Preterm newborn OR Preterm newborns OR Prematurity OR Stillbirth OR Stillbirths OR Still Birth OR Still Births OR Still Born Baby OR Still Born Babies OR Low Birth Weight OR Low Birth Weights OR Low-Birth-Weight OR Very Low Birth Weight OR Very-Low-Birth-Weight OR Very Low Birth Weight OR Extremely Low Birth Weight OR Congenital Abnormality OR Deformity OR Deformities OR Congenital Defect OR Congenital Defects OR Birth Defect OR Birth Defects OR Fetal Malformation OR Fetal Malformations OR Fetal Anomaly OR Fetal Anomalies OR Congenital Anomaly OR Congenital Deformity OR Development Anomaly OR Malformation OR Malformative Disease OR Congenital Malformation OR Perinatal Death OR Perinatal Deaths OR Neonatal Death OR Neonatal Deaths OR New Born Death OR New Born Deaths OR Cumulative clinical pregnancy OR Cumulative clinical pregnancies OR Cumulative clinical pregnancy rate OR Cumulative Live Birth Rate	191 566
4	randomized controlled trail OR randomized OR placebo OR randomised OR random OR RCT	522 627
5	Step1 AND Step2 AND Step3 AND Step4	**306**

Human Reproduction, 2026, 1–1
https://doi.org/10.1093/humrep/deag066
Supplementary Data

知网
On 10 June 2025
Published by 31 May 2025

Step	Searches (Professional search)	Results
1	SU%=（‘体外受精’+‘IVF’） AND SU%=（‘卵细胞浆内单精子注射’+‘卵胞浆内单精子注射’ +‘ICSI’） AND FT%=（‘活产率’ + ‘完全受精失败’ + ‘受精率’ + ‘植入率’+ ‘临床妊娠率’ + ‘持续妊娠’ + ‘自发性流产’ + ‘早产’ + ‘死产’ + ‘低出生体重’ + ‘出生缺陷’ + ‘先天畸形’ + ‘先天性异常’ + ‘先天性缺陷’ + ‘新生儿死亡’） AND AB%=（‘随机对照’ + ‘随机’ + ‘RCT’）	**207**

万方
On 10 June 2025
Published by 31 May 2025

Step	Searches (Professional search)	Results
1	主题：（体外受精 or IVF）and 主题：（卵胞浆内单精子注射 or 卵细胞浆内单精子注射 or ICSI）and 主题：（活产率 or 完全受精失败 or 受精率 or 植入率 or 临床妊娠率 or 持续妊娠 or 自发性流产 or 早产 or 死产 or 低出生体重 or 出生缺陷 or 先天畸形 or 先天性异常 or 先天性缺陷 or 新生儿死亡）and 主题：（随机对照 or 随机 or RCT）	**348**

Bold values in the table indicate the number of extracted records.
