# Supplementary material for: Does intracytoplasmic sperm injection outperform conventional in vitro fertilization in couples without severe male factor infertility? A systematic review and meta-analysis of randomized controlled trials
Source: Hum Reprod. 2026 May 22;41(7):1173–82. doi: 10.1093/humrep/deag066 (PMC13334920; doi:10.1093/humrep/deag066)
Supplement: deag066_Supplementary_Table_S2 [file deag066_supplementary_table_s2.pdf]

Human Reproduction, 2026, 1–3
https://doi.org/10.1093/humrep/deag066
Supplementary Data

human reproduction
OXFORD

Supplementary Table S2. Detailed information of the included studies.

Study ID	Duration of infertility	Age (years)	Inclusion criteria	Exclusion criteria	Ovarian stimulation
Bhattacharya *et al.* (2001)	IVF: 57.3 ± 29.0 months<br>ICSI: 57.4 ± 28.0 months	IVF:<br>33.7 ± 4.8 (male)<br>30.9 ± 4.1 (female)<br>ICSI:<br>33.3 ± 5.1 (male)<br>31.6 ± 3.2 (female)	Eligible couples were awaiting IVF for an indication other than severe male-factor infertility. The inclusion criteria were age of the female partner below 37 years and minimum acceptable semen characteristics, including sperm density of 20 million/mL, progressive motility of 40%, and acceptable morphology (as defined by local laboratory standards in each center) to allow consideration of IVF. Criteria for normal morphology varied between 10% and 20% in the different centers, according to local laboratory standards. When morphology was abnormal but was above a cut-off value of 4% (in the presence of normal motility and density), a diagnosis of mild male infertility was made, and these couples were included in the study because they would generally be offered IVF rather than ICSI treatment.	Couples were excluded from the study if the fertilization rate in a previous IVF cycle was <20%, baseline serum concentration of FSH in the female partner was more than 12 IU/l, or if the couple had undergone three or more previous IVF treatment cycles. Couples were also excluded if semen characteristics were deemed sufficiently abnormal (according to local protocol) to warrant ICSI rather than IVF treatment.	Long protocol
Foong *et al.* (2006)	IVF: 57.2 ± 35.5 months<br>ICSI: 64.5 ± 28.2 months	IVF: 33.0 ± 3.6<br>ICSI: 33.7 ± 2.1	(i) Female age 18–40 years; (ii) regular ovulatory menstrual cycles; (iii) Day #3 estradiol (E2) < 200 pmol/l, FSH < 15 IU/l, LH < 8 IU/l, normal thyroid-stimulating hormone; (iv) ≥3 previous IUI cycles with clomiphene citrate or gonadotropins; (v) normal uterine	Not reported	GnRH agonist

(continued)

Supplementary Table S2. Continued

Study ID	Duration of infertility	Age (years)	Inclusion criteria	Exclusion criteria	Ovarian stimulation
			cavity, fallopian tubes, and presence of both ovaries, normal ultrasound (US), and previous laparoscopy excluding Stage III or IV endometriosis; (vi) all male partners had a normal semen analysis by WHO criteria.		
Dang *et al.* (2021)	IVF: 4.0 (2.0–6.0) years<br>ICSI: 3.0 (2.0–5.0) years	IVF:<br>35.3 ± 5.6 (male)<br>32.6 ± 4.7 (female)<br>ICSI: 35.2 ± 5.2 (male)<br>32.7 ± 4.6 (female)	Eligible couples were aged at least 18 years, and the male partner's sperm count and motility (progressive motility) were normal based on WHO 2010 criteria (total sperm count $\geq 39 \times 10^6$ sperm, progressive motility ≥32%). Couples had to have undergone two or fewer previous conventional IVF or ICSI attempts, have used an antagonist protocol for ovarian stimulation, and agree to have two or fewer embryos transferred, and not simultaneously be participating in other IVF trials.	Couples undergoing *in-vitro* maturation cycles, couples using frozen semen, or couples with poor fertilization (≤25%) in a previous cycle were excluded.	FSH and gonadotropin releasing antagonist protocol
Fancsovits *et al.* (2023)	Not reported	IVF: 40.4 ± 3.5<br>ICSI: 40.1 ± 4.0	(i) ≥40 years of female age and/or ≤4 oocytes collected; (ii) patients with normal semen parameters or non-severe male factor infertility.	(i) The fertilization rate in a previous IVF treatment was <50%; (ii) cycles with surgical sperm retrieval; (iii) sperm or oocyte donation; (iv) preimplantation genetic testing.	GnRH agonist long protocol
Wang *et al.* (2024)	IVF: 3 (2–5) years<br>ICSI: 3 (2–5) years	IVF:<br>34 (31–38) (male)<br>33 (30–37) (female)<br>ICSI: 34 (31–38) (male)<br>34 (31–37) (female)	Couples with infertility were eligible if they were scheduled for their first or second IVF or ICSI cycle following a GnRH agonist or antagonist protocol as their controlled ovarian hyperstimulation treatment; the male partner showed non-severe male factor during initial evaluation. Non-severe male	Couples were ineligible if they were treated with donor sperm or donor eggs, underwent preimplantation genetic testing, used frozen semen, or had poor fertilization in a previous IVF cycle (≤25%). Furthermore, couples in whom no oocytes were retrieved and couples with a total	GnRH agonist protocol or GnRH antagonist protocol

(continued)

Supplementary Table S2. Continued

Study ID	Duration of infertility	Age (years)	Inclusion criteria	Exclusion criteria	Ovarian stimulation
			factor was defined as oligoasthenozoospermia (sperm concentration 5–15 × 10^6 sperm per ml and progressive motility 10–32%), oligozoospermia (sperm concentration 5–15 × 10^6 sperm per ml and progressive motility ≥32%), or asthenozoospermia (sperm concentration ≥15 × 10^6 sperm per ml and progressive motility 10–32%), according to the fifth edition of WHO's laboratory manual for the examination and processing of human semen. Teratospermia was defined as sperm with <4% typical morphology.	progressive motile sperm count for insemination of <0.1 × 10^6 sperm per ml on the day of oocyte retrieval (i.e. the number of progressive motile sperm for insemination was not sufficient for typical fertilization in the conventional IVF procedure) were not eligible.	
Berntsen *et al.* (2025)	IVF: 26 (21-36) months<br>ICSI: 26 (20-36) months	IVF:<br>34.6 ± 6.0 (male)<br>33.5 ± 4.4 (female)<br>ICSI:<br>34.2 ± 5.7 (male)<br>33.0 ± 4.6 (female)	Eligible participants were women aged 18–42 years with no prior c-IVF or ICSI treatment and a body mass index of 18–35kg/m^2. Participants were considered eligible if they qualified for c-IVF treatment, which required male partner sperm containing a minimum of 2 million progressively motile spermatozoa (after density gradient purification, wash steps and resuspension), or if donor sperm was to be used.	Women with severe comorbidity (including ovarian cysts >4 cm, liver or kidney disease, unregulated thyroid disease, endometriosis Stages 3–4, hypogonadotropic hypogonadism, or other severe comorbidities such as diabetes or cardiovascular disease) and couples/women using donor oocytes or frozen oocytes were not included.	GnRH-antagonist protocol or a long GnRH-agonist protocol
