# Supplementary material for: Does intracytoplasmic sperm injection outperform conventional in vitro fertilization in couples without severe male factor infertility? A systematic review and meta-analysis of randomized controlled trials
Source: Hum Reprod. 2026 May 22;41(7):1173–82. doi: 10.1093/humrep/deag066 (PMC13334920; doi:10.1093/humrep/deag066)
Supplement: deag066_Supplementary_Table_S3 [file deag066_supplementary_table_s3.pdf]

Human Reproduction, 2026, 1–1
https://doi.org/10.1093/humrep/deag066
Supplementary Data

human reproduction
OXFORD

Supplementary Table S3. Outcomes extracted from included studies.

Study ID	Live birth	Cumulative live birth	Clinical pregnancy	Fertilization	Total fertilization failure	Implantation	Ongoing pregnancy	Miscarriage	Stillbirth	Preterm birth	Low birth weight	Birth defect	Neonatal death	Multiple pregnancy	Ectopic pregnancy	Gestational diabetes	Gestational hypertension
Bhattacharya *et al.* (2001)	×	×	√	×	×	√	×	×	×	×	×	×	×	√	×	×	×
Foong *et al.* (2006)*	√	×	√	×	√	√	×	×	×	×	×	×	×	×	×	×	×
Dang *et al.* (2021)	√	√	√	√	√	√	√	√	×	√	√	√	×	√	√	√	√
Fancsovits *et al.* (2023)†	×	×	×	×	×	√	×	×	×	×	×	×	×	×	×	×	×
Wang *et al.* (2024)	√	√	√	√	√	√	√	√	×	√	√	√	√	√	√	√	√
Berntsen *et al.* (2025)	√	√	√	√	√	√	√	√	√	√	√	√	×	√	√	√	√

* Fertilization rate was not extracted from this study because of the unclear number of oocytes.
† The live birth rate, clinical pregnancy rate, total fertilization failure rate, and multiple pregnancy rate were not extracted because the number of participants in each group was not reported.
