# Supplementary material for: Does intracytoplasmic sperm injection outperform conventional in vitro fertilization in couples without severe male factor infertility? A systematic review and meta-analysis of randomized controlled trials
Source: Hum Reprod. 2026 May 22;41(7):1173–82. doi: 10.1093/humrep/deag066 (PMC13334920; doi:10.1093/humrep/deag066)
Supplement: deag066_Supplementary_Table_S4 [file deag066_supplementary_table_s4.pdf]

*Human Reproduction*, 2026, 1–2
https://doi.org/10.1093/humrep/deag066
**Supplementary Data**

human reproduction

OXFORD

**Supplementary Table S4.** Definitions of other secondary outcomes extracted from included studies.

| Outcomes | Definitions |
|---|---|
| **Live birth rate** | The number of couples who achieved a live birth after the first embryo transfer/number of couples × 100<br>• Foong *et al.* (2006) did not provide put clear definition in terms of gestation period.<br>• In Dang *et al.* (2021), live birth was defined as the birth of at least one baby after 24 weeks of gestation that showed any sign of life, such as respiration, heartbeat, umbilical pulsation, or movement of voluntary muscles (twins as a single count).<br>• In Wang *et al.* (2024), live birth was defined as the birth of at least one baby after 22 weeks of gestation who showed any evidence of life.<br>• In Berntsen *et al.* (2025), live birth was defined as the delivery of one or more living infants at or beyond 22 weeks of gestation. |
| **Cumulative live birth rate** | The number of couples who achieved a live birth after predefined time period/number of couples × 100<br>• Dang *et al.* (2021) reported the cumulative live birth rate of 12 months after random allocation.<br>• Wang *et al.* (2024) reported the cumulative live birth rate of embryos transferred within 12 months of randomization.<br>• Berntsen *et al.* (2025) reported the cumulative live birth rate of 12 months after the inclusion of the last participant. The cumulative live birth rate of 12 months after randomization was obtained from the authors. |
| **Clinical pregnancy rate** | The number of couples with at least one gestational sac or definitive clinical signs of pregnancy confirmed by ultrasonography after the first embryo transfer/number of couples × 100<br>• In Bhattacharya *et al.* (2001), a clinical pregnancy was diagnosed in the presence of fetal heart activity shown by transvaginal ultrasonography. The timing of ultrasonography for clinical pregnancy confirmation was not provided.<br>• In Foong *et al.* (2006), clinical pregnancy was defined as the positive cardiac activity on ultrasonography at 6–7 weeks gestation.<br>• In Dang *et al.* (2021), clinical pregnancy was defined as the presence of at least one gestational sac on ultrasound at 7 weeks' gestation with the detection of heartbeat activity.<br>• In Wang *et al.* (2024), clinical pregnancy was defined as one or more observed gestational sac or definitive clinical signs of pregnancy under ultrasonography at 7 weeks of gestation (including clinically documented ectopic pregnancy).<br>• In Berntsen *et al.* (2025), ongoing pregnancy, which is identified by fetal heartbeat on ultrasound in gestational Week 7–8, was in corresponded to clinical pregnancy in the other studies. |
| **Fertilization rate** | The number of zygotes with two pronuclei/number of oocytes retrieved in per woman/couple |
| **Total fertilization failure rate** | The number of couples who underwent total fertilization failure, which was defined as no oocyte formed 2PN in this given cycle/number of couples × 100 |
| **Implantation rate** | The number of gestational sacs after the first embryo transfer/number of embryos transferred × 100<br>• In Bhattacharya *et al.* (2001), implantation rate was calculated as the proportion of gestational sacs visible on ultrasonography per embryo replaced.<br>• In Foong *et al.* (2006), implantation rate was defined as the number of gestational sacs on ultrasonography at 6–7 weeks gestation per number of embryos transferred.<br>• In Fancsovits *et al.* (2023), implantation rate was defined as the number of gestational sacs divided by the number of embryos transferred.<br>• In Dang *et al.* (2021), implantation rate was defined as the number of gestational sacs per number of embryos transferred 3 weeks after the first transfer.<br>• In Wang *et al.* (2024), implantation rate was defined as the number of gestational sacs observed per embryo transferred.<br>• Berntsen *et al.* (2025) calculated implantation rate using number of participants who had a positive serum human chronic gonadotropin (s-hCG) test after first transfer as numerator (ectopic pregnancies and anembyonic pregnancies were excluded) |
| **Ongoing pregnancy rate** | The number of couples who achieved ongoing pregnancy, which was defined as the presence of a gestational sac and or fetal heartbeat after 12 weeks of gestation or beyond, after the first embryo transfer/number of couples ×100<br>• Ongoing pregnancy (GA 12) in Berntsen *et al.* (2025) was obtained from the original investigators. |

(continued)

**Supplementary Table S4.** Continued

| Outcomes | Definitions |
| --- | --- |
| **Miscarriage rate** | The number of couples who underwent miscarriage after the first embryo transfer/number of couples ×100<br>• In Dang *et al.* (2021), miscarriage was complete loss of clinical pregnancy at 24 weeks of gestation.<br>• In Wang *et al.* (2024), miscarriage was the spontaneous loss of an intra-uterine pregnancy prior to 22 completed weeks of gestational age.<br>• In Berntsen *et al.* (2025), miscarriage included 'early pregnancy loss (< GA 12)' and 'late pregnancy loss (GA 11 + 6–22 + 0)'. |
| **Stillbirth rate** | The number of couples who underwent stillbirth after the first embryo transfer/number of couples ×100 |
| **Preterm birth rate** | The number of couples who underwent preterm birth after the first embryo transfer/number of couples ×100<br>• In Dang *et al.* (2021), preterm birth was defined as any delivery at < 24, < 28, < 32, < 37 completed weeks' gestation. The data of < 37 completed weeks' gestation was extracted.<br>• In Wang *et al.* (2024), preterm birth was defined as birth of a fetus delivered after 22 and before 37 completed weeks of gestational age in participants confirmed ongoing pregnancy.<br>• In Berntsen *et al.* (2025), preterm birth was before GA 37 + 0. |
| **Low birth weight rate** | The number of couples whose offspring weighted < 2500 g at birth after the first embryo transfer/number of couples ×100 |
| **Birth defect rate** | The number of couples whose offspring had birth defect after the first embryo transfer/number of couples ×100<br>• In Dang *et al.* (2021), any congenital anomaly will be included at birth.<br>• In Wang *et al.* (2024), birth defect was defined as structural or functional disorders that occur during intrauterine life and can be identified prenatally, at birth or later in life), including trisomy 13, 18, 21, neural tube defect, congenital heart disease, cleft lip, excessive numbers of fingers or toes, hydrocephalus.<br>• Berntsen *et al.* (2025) did not provide clear definition. |
| **Neonatal death rate** | The number of couples whose live born baby underwent death within 28 days of birth after the first embryo transfer/number of couples ×100 |
| **Multiple pregnancy rate** | The number of couples with multiple pregnancy after the first embryo transfer/number of couples ×100<br>• Foong *et al.* (2006) did not provide clear definition.<br>• In Dang *et al.* (2021), multiple pregnancy rate was explained as two or more gestational sacs or positive heart beats by transvaginal sonography 5 weeks after embryo placement.<br>• In Wang *et al.* (2024), multiple pregnancy was defined as a pregnancy with two or more gestational sacs or positive heart beats at 7 weeks of gestation.<br>• Berntsen *et al.* (2025) included twin pregnancy. |
| **Ectopic pregnancy rate** | The number of couples with ectopic pregnancy after the first embryo transfer/number of couples ×100<br>• In Dang *et al.* (2021), ectopic pregnancy was ectopic nidation of a pregnancy confirmed by sonography or laparoscopy at 12 weeks of gestation.<br>• In Wang *et al.* (2024), ectopic pregnancy was defined as the implantation takes place outside the uterine cavity, confirmed by sonography or laparoscopy.<br>• Berntsen *et al.* (2025) did not provide clear definition. |
| **Gestational diabetes rate** | The number of couples who developed gestational diabetes during pregnancy after the first embryo transfer/number of couples ×100 |
| **Gestational hypertension rate** | The number of couples who developed gestational hypertension rate during pregnancy after the first embryo transfer/number of couples ×100<br>• In Dang *et al.* (2021), hypertensive disorders of pregnancy included pregnancy induced hypertension, pre-eclampsia, and eclampsia at birth.<br>• In Wang *et al.* (2024), hypertensive disorders of pregnancy (comprising pregnancy-induced hypertension, pre-eclampsia, and eclampsia)<br>• Berntsen *et al.* (2025) reported gestational hypertension |
