# Supplementary material for: Does intracytoplasmic sperm injection outperform conventional in vitro fertilization in couples without severe male factor infertility? A systematic review and meta-analysis of randomized controlled trials
Source: Hum Reprod. 2026 May 22;41(7):1173–82. doi: 10.1093/humrep/deag066 (PMC13334920; doi:10.1093/humrep/deag066)
Supplement: deag066_Supplementary_Table_S6 [file deag066_supplementary_table_s6.pdf]

Human Reproduction, 2026, 1–2
https://doi.org/10.1093/humrep/deag066
Supplementary Data

human reproduction

OXFORD

Supplementary Table S6. Summary of findings (SoF) tables.

Outcomes	Estimated risks/means		Relative effect (95% CI)	No. of Participants (studies)	Quality of evidence
**Main analyses**					
Live birth rate	739 per 2145	699 per 2130	RR 0.96 (0.85–1.09)	4275 (4 RCTs)	High ⊕⊕⊕⊕
Cumulative live birth rate	1003 per 2115	908 per 2100	RR 0.92 (0.84–1.01)	4215 (3 RCTs)	High ⊕⊕⊕⊕
Clinical pregnancy rate	929 per 2358	878 per 2332	RR 0.96 (0.88–1.04)	4690 (5 RCTs)	High ⊕⊕⊕⊕
Total fertilization failure rate	107 per 2145	91 per 2130	RR 0.86 (0.65–1.13)	4275 (4 RCTs)	High ⊕⊕⊕⊕
Fertilization rate	NA	NA	MD −0.01 (−0.05 to 0.03)	4215 (3 RCTs)	Moderate ⊕⊕⊕○ due to inconsistency
Implantation rate	1216 per 3643	1120 per 3615	RR 0.93 (0.86–1.01)	–(6 RCTs)	High ⊕⊕⊕⊕
Ongoing pregnancy rate	745 per 2115	706 per 2100	RR 0.96 (0.85–1.08)	4215 (3 RCTs)	High ⊕⊕⊕⊕
Miscarriage rate	92 per 2115	109 per 2100	RR 1.19 (0.91–1.56)	4215 (3 RCTs)	High ⊕⊕⊕⊕
Stillbirth rate	1 per 408	0 per 414	RR 0.33 (0.01–8.04)	826 (1 RCT)	High ⊕⊕⊕⊕
Preterm birth rate	126 per 2115	96 per 2100	RR 0.77 (0.59–1.00)	4215 (3 RCTs)	High ⊕⊕⊕⊕
Low birth weight rate	61 per 2115	67 per 2100	RR 1.10 (0.78–1.56)	4215 (3 RCTs)	High ⊕⊕⊕⊕
Birth defect rate	24 per 2115	15 per 2100	RR 0.63 (0.33–1.20)	4215 (3 RCTs)	High ⊕⊕⊕⊕
Neonatal death rate	1 per 1175	2 per 1154	RR 2.04 (0.18–22.43)	2329 (1 RCT)	Moderate ⊕⊕⊕○ due to imprecision
Multiple pregnancy rate	208 per 2328	177 per 2302	RR 0.86 (0.71–1.04)	4630 (4 RCTs)	High ⊕⊕⊕⊕
Ectopic pregnancy rate	28 per 2115	23 per 2100	RR 0.83 (0.48–1.43)	4215 (3 RCTs)	High ⊕⊕⊕⊕
Gestational diabetes rate	69 per 2115	66 per 2100	RR 0.96 (0.69–1.34)	4215 (3 RCTs)	High ⊕⊕⊕⊕
Gestational hypertension rate	37 per 2115	27 per 2100	RR 0.73 (0.45–1.20)	4215 (3 RCTs)	High ⊕⊕⊕⊕
**Subgroup analyses**					
**Normal or non-severe male factor infertility**					
Live birth rate	559 per 1583	500 per 1568	RR 0.91 (0.82–1.00)	3151 (2 RCTs)	High ⊕⊕⊕⊕
Cumulative live birth rate	786 per 1583	686 per 1568	RR 0.88 (0.82–0.95)	3151 (2 RCTs)	High ⊕⊕⊕⊕
Total fertilization failure rate	71 per 1583	62 per 1568	RR 0.94 (0.56–1.57)	3151 (2 RCTs)	High ⊕⊕⊕⊕
Fertilization rate	NA	NA	MD −0.03 (−0.09 to 0.03)	3151 (2 RCTs)	Moderate ⊕⊕⊕○ due to inconsistency
Implantation rate	772 per 1583	703 per 1568	RR 0.92 (0.85–1.00)	3151 (2 RCTs)	High ⊕⊕⊕⊕
Ongoing pregnancy rate	571 per 1583	516 per 1568	RR 0.91 (0.83–1.01)	3151 (2 RCTs)	High ⊕⊕⊕⊕
Miscarriage rate	64 per 1583	82 per 1568	RR 1.29 (0.94–1.78)	3151 (2 RCTs)	High ⊕⊕⊕⊕
Preterm birth rate	91 per 1583	70 per 1568	RR 0.78 (0.58–1.06)	3151 (2 RCTs)	High ⊕⊕⊕⊕
Low birth weight rate	55 per 1583	57 per 1568	RR 1.05 (0.73–1.51)	3151 (2 RCTs)	High ⊕⊕⊕⊕
Birth defect rate	21 per 1583	13 per 1568	RR 0.62 (0.31–1.24)	3151 (2 RCTs)	High ⊕⊕⊕⊕
Multiple pregnancy rate	125 per 1583	104 per 1568	RR 0.85 (0.66–1.08)	3151 (2 RCTs)	High ⊕⊕⊕⊕
Ectopic pregnancy rate	18 per 1583	13 per 1568	RR 0.73 (0.36–1.48)	3151 (2 RCTs)	High ⊕⊕⊕⊕
Gestational diabetes rate	42 per 1583	41 per 1568	RR 0.98 (0.64–1.50)	3151 (2 RCTs)	High ⊕⊕⊕⊕
Gestational hypertension rate	36 per 1583	26 per 1568	RR 0.73 (0.44–1.20)	3151 (2 RCTs)	High ⊕⊕⊕⊕
**Non-male factor infertility with unspecified type**					
Live birth rate	166 per 532	184 per 532	RR 1.11 (0.93–1.32)	1064 (1 RCT)	Moderate ⊕⊕⊕○ due to indirectness
Clinical pregnancy rate	284 per 745	280 per 734	RR 0.94 (0.69–1.28)	1479 (2 RCTs)	Very low ⊕○○○ due to risk of bias, inconsistency, and indirectness
Implantation rate	373 per 1271	356 per 1296	RR 0.88 (0.66–1.18)	1479 (2 RCTs)	Very low ⊕○○○ due to risk of bias, inconsistency, and indirectness
Multiple pregnancy rate	83 per 745	73 per 734	RR 0.89 (0.66–1.20)	1479 (2 RCTs)	Low ⊕⊕○○ due to risk of bias and indirectness

(continued)

Supplementary Table S6. Continued

Outcomes	Estimated risks/means		Relative effect (95% CI)	No. of Participants (studies)	Quality of evidence
**Tubal factor infertility**					
Live birth rate	31 per 120	45 per 134	RR 1.28 (0.89–1.83)	254 (1 RCT)	High ⊕⊕⊕⊕
**Endometriosis**					
Live birth rate	3 per 16	8 per 20	RR 2.13 (0.67–6.76)	36 (1 RCT)	Low ⊕⊕○○ due to imprecision
**Low oocyte number**					
Implantation rate	27 per 250	15 per 208	RR 0.67 (0.37–1.22)	– (1 RCT)	High ⊕⊕⊕⊕
**Advanced maternal age**					
Implantation rate	24 per 150	16 per 146	RR 0.68 (0.38–1.24)	– (1 RCT)	High ⊕⊕⊕⊕
**Unexplained infertility**					
Live birth rate	79 per 213	88 per 229	RR 1.04 (0.82–1.32)	442 (2 RCTs)	Moderate ⊕⊕⊕○ due to risk of bias
Clinical pregnancy rate	15 per 30	15 per 30	RR 0.87 (0.50–1.49)	60 (1 RCT)	Moderate ⊕⊕⊕○ due to imprecision
Total fertilization failure rate	2 per 30	0 per 30	RR 0.20 (0.01–4.00)	60 (1 RCT)	Low ⊕⊕○○ due to imprecision
Implantation rate	31 per 81	32 per 72	RR 1.16 (0.80–1.70)	60 (1 RCT)	Moderate ⊕⊕⊕○ due to imprecision

RR, risk ratio; RCT, randomized controlled trial; MD, mean difference.

© The Author(s) 2026. Published by Oxford University Press on behalf of European Society of Human Reproduction and Embryology.
This is an Open Access article distributed under the terms of the Creative Commons Attribution License (https://creativecommons.org/licenses/by/4.0/), which permits unrestricted reuse, distribution, and reproduction in any medium, provided the original work is properly cited.
Human Reproduction, 2026, 00, 1–10
https://doi.org/10.1093/humrep/deag066
Meta-Analysis
